# DP1 receptor signaling prevents the onset of intrinsic apoptosis in eosinophils and functions as a transcriptional modulator

**DOI:** 10.1002/JLB.3MA1017-404R

**Published:** 2018-04-01

**Authors:** Miriam Peinhaupt, David Roula, Anna Theiler, Miriam Sedej, Rudolf Schicho, Gunther Marsche, Eva M. Sturm, Ian Sabroe, Marc E. Rothenberg, Akos Heinemann

**Affiliations:** ^1^ Otto Loewi Research Center for Vascular Biology Immunology and Inflammation Division of Pharmacology Medical University of Graz Graz Austria; ^2^ BioTechMed‐Graz Graz Austria; ^3^ Department of Infection Immunity and Cardiovascular Disease University of Sheffield Sheffield England; ^4^ Division of Allergy and Immunology Cincinnati Children's Hospital Medical Center Cincinnati Ohio USA

**Keywords:** allergy, apoptosis, inflammation, prostanoids

## Abstract

Prostaglandin (PG) D_2_ is the ligand for the G‐protein coupled receptors DP1 (D‐type prostanoid receptor 1) and DP2 (also known as chemoattractant receptor homologous molecule, expressed on Th2 cells; CRTH2). Both, DP1 and DP2 are expressed on the cellular surface of eosinophils; although it has become quite clear that PGD_2_ induces eosinophil migration mainly via DP2 receptors, the role of DP1 in eosinophil responses has remained elusive. In this study, we addressed how DP1 receptor signaling complements the pro‐inflammatory effects of DP2. We found that PGD_2_ prolongs the survival of eosinophils via a DP1 receptor‐mediated mechanism that inhibits the onset of the intrinsic apoptotic cascade. The DP1 agonist BW245c prevented the activation of effector caspases in eosinophils and protected mitochondrial membranes from depolarization which—as a consequence—sustained viability of eosinophils. DP1 activation in eosinophils enhanced the expression of the anti‐apoptotic gene BCL‐X_L_, but also induced pro‐inflammatory genes, such as VLA‐4 and CCR3. In HEK293 cells that overexpress recombinant DP1 and/or DP2 receptors, activation of DP1, but not DP2, delayed cell death and stimulated proliferation, along with induction of serum response element (SRE), a regulator of anti‐apoptotic, early‐response genes. We conclude that DP1 receptors promote the survival via SRE induction and induction of pro‐inflammatory genes. Therefore, targeting DP1 receptors, along with DP2, may contribute to anti‐inflammatory therapy in eosinophilic diseases.

## Abstract 2

The PGD2‐DP1 axis impedes caspase activity, protects mitochondrial function and consequently sustains eosinophil survival by controlling SRE induction and upregulation of pro‐inflammatory and anti‐apoptotic genes.

**Abbreviations**
Δ^12^‐PGJ_2_: Δ12‐prostaglandin J2
15‐deoxy‐PGJ_2_: 15‐deoxy‐Δ12,14‐ prostaglandin J2
Bcl‐X_L_: B‐cell lymphoma‐extra‐large
BLT1/2: leukotriene B4 receptor 1/2
C5A‐R1: complement component C5a receptor 1
CCCP: carbonyl cyanide m‐chlorophenyl hydrazon
CCR3: C‐C chemokine receptor type 3
DKPGD_2_: 13,14di‐hydro 15‐keto PGD_2_
DP1: D‐type prostanoid receptor 1
DP2: D‐type prostanoid receptor 2 [also chemoattractant receptor homologous molecule of Th2 cells (CRTH2)]
ECIS: electric cell‐substrate impedance sensing
HEK293: human embryonic kidney cells 293
PG: prostaglandin
PI: propidium iodide
PPAR: peroxisome proliferator‐activated receptor
SRE: serum response element
SRF: serum response factor
VLA‐4: very late antigen 4

## 1 INTRODUCTION

Numerous factors drive the progression of allergic conditions, affecting either the immediate, early, or the late phase of the allergic response. In the pivotal step of the inflammatory cascade mast cells release mediators such as histamine, prostaglandin (PG) D_2_, leukotriene C4, TNF‐α, and many others,^1^ which start off the allergic response in the first place, induce the recruitment of inflammatory cells into the tissue and finally stimulate the surrounding and infiltrating cells to drive the transition from early to late phase, resulting in tissue damage. Eosinophils are considered as crucial effector cells in chronic allergic inflammation since they are involved in increasing epithelial‐to‐mesenchymal transition,^2^, ^3^ thickening of airway walls, airway hyperresponsiveness, and angiogenesis.^4^, ^5^ Therefore, eosinophils are of major therapeutic interest in allergic diseases.

Concomitant with allergic inflammation, the life span of immune cells such as macrophages, Th2 cells, or eosinophils is known to be prolonged.^6^ Accordingly, blocking the prominent pro‐survival cytokine IL‐5 using the monoclonal antibodies mepolizumab or reslizumab profoundly reduced eosinophil numbers and effectively prevented exacerbations in cases of severe, highly eosinophilic asthma.^7^, ^8^, ^9^ This demonstrated that targeting mechanisms that regulate eosinophil survival is a clinically relevant approach in allergic diseases.

PGD_2_ is the major lipid mediator released by mast cells following crosslinking of allergen‐specific IgE molecules displayed on their surface in sensitized individuals as part of the early allergic reaction.^10^, ^11^ Furthermore, eosinophils, dendritic cells, macrophages, and endothelial cells^12^ produce PGD_2_ and substantial amounts have been detected in tissues affected by allergic reactions, such as lung,^13^ skin,^14^, ^15^ and esophagus_._ 16 PGD_2_ is the ligand for two 7‐transmembrane G protein‐coupled receptors, named D‐type prostanoid (DP) receptor 1 and chemoattractant receptor homologous molecule expressed on Th2 cells (CRTH2, also termed DP2),^17^ that are co‐expressed on the cell surface of eosinophils.^18^ Although DP1 and DP2 bind PGD_2_ with similar avidity,^17^ DP2 shares greater homology with classical chemoattractant receptors (e.g., BLT1/2, C5a‐R1, CCR3, FPR) than with other prostanoid receptors.^19^, ^20^

PGD_2_ and selective DP2 agonists cause chemotaxis and pro‐inflammatory activation of eosinophils including cellular responses, such as Ca^2+^ flux, CD11b upregulation, respiratory burst, and eosinophil cationic protein release,^20^, ^21^, ^22^, ^23^ via binding to DP2. In contrast, the functional responses and exact signaling pathways triggered by DP1 activation in eosinophils have remained unclear up to now. We have previously shown in eosinophils and heterologous expression systems that the DP1 receptor augments the signaling and functionality of the DP2 receptor, including intracellular Ca^2+^ flux, chemotaxis, and oxidative burst.^18^, ^24^, ^25^ Defining the role of DP1 on eosinophils might hence contribute to the full understanding of the role of PGD_2_ in allergic disease and asthma and explain the limited efficacy of DP2 antagonists in clinical studies.^12^ One single report described that activation of DP1 on eosinophils protected them from undergoing apoptosis^21^; whether this resulted in extended life‐span of eosinophils and was due to activation of pro‐survival signaling pathways has still remained unresolved. Another study, however, presented opposing data arguing for a pro‐apoptotic effect of higher concentrations (10 μM) of PGD_2_ that was attributed to inhibition of IkB degradation in eosinophils by the PGD_2_ metabolites 15‐deoxy‐PGJ_2_ and Δ^12^‐PGJ_2_.^26^

In order to unequivocally decipher the role of PGD_2_ and its receptors in the regulation of eosinophil survival, this study investigated how DP1 regulates eosinophil survival and operates as a transcriptional regulator in a pro‐inflammatory setting. Thus, we propose that DP1 antagonists could have beneficial effects by accelerating the resolution of allergic inflammation.

## 2 METHODS

### 2.1 Materials

Prostaglandin D_2_ receptor agonists PGD_2_, BW245c, 13,14‐di‐hydro 15‐keto PGD_2_ (DK‐PGD_2_), and antagonists MK0524, Cay10471, and BW A868c were purchased from Cayman, Ann Arbor, USA, and rosiglitazone, BH3I‐1, and HA14.1 were purchased from Sigma–Aldrich, St. Louis, USA.

### 2.2 Preparation of human peripheral blood eosinophils

Human eosinophils were isolated from blood samples of healthy volunteers according to a protocol approved by the Institutional Review Board of the Medical University of Graz or Cincinnati Children's Hospital Medical Center as previously described.^27^ In brief, erythrocytes were removed by dextran sedimentation and polymorphonuclear leukocytes were separated from mononuclear cells by density gradient centrifugation (Histopaque 1077, Sigma–Aldrich). Eosinophils were separated from neutrophils in the polymorphonuclear leukocyte fraction by negative magnetic selection using the MACS cell separation system (Eosinophil Isolation Kit, Milteny Biotech, Bergisch Gladbach, Germany) with a resulting purity of typically ≥98%.

### 2.3 Human embryonic kidney cells

Previously described Human embryonic kidney 293 (HEK293) cells lines stably expressing DP1, DP2, or both receptors^18^ are referred to as HEK‐DP1, HEK‐DP2+DP1, and HEK‐DP2. HEK293 cells were kept in serum containing selection medium (DMEM + 10% FBS) with either neomycin (0.2%) or zeocin (0.4%), or both. Cells were propagated in 75 cm^2^ cell culture flasks and medium was changed every second day. Cells were harvested after 3–4 days of culturing when they have grown to 90% confluence. Zeocin and Neomycin (Geneticin, G418), DMEM, and FBS were purchased from Thermo Fisher Scientific, Waltham, MA, USA.

### 2.4 Reporter gene assays

HEK‐DP1, HEK‐DP2, or HEK‐DP1 + DP2 cells were seeded in 96‐well plates at a density of 6 × 10^5^ cells/well and grown to approximately 90% confluence in non‐selective medium (DMEM + 10% FCS). Using Lipofectamine 2000 (Invitrogen, Carlsbad, CA, USA) cells were transiently transfected with pSRE‐Luc reporter plasmid (100 ng/well) according to the manufacturer's protocol. Twenty‐four hours post transfection cells were pre‐treated with antagonists for 20 min (or the respective vehicle) followed by agonist stimulation for 3 h. Luciferase activity was determined using the Steadylite Plus Kit (PerkinElmer, Waltham, USA), as previously described.^28^, ^29^ Chemiluminescence was measured on a TopCount NXT device (Perkin Elmer/Packard Bioscience).

### 2.5 Annexin V/propidium iodide co‐staining

Isolated eosinophils (5 × 10^5^/ml) were kept in RPMI (Thermo Fisher Scientific) supplemented with 1% FBS and PenStrep (Sigma–Aldrich) and stimulated with 1 μM PGD_2_, DK‐PGD_2_, or BW245c for 18 h at 37°C. After washing, cells were incubated with Annexin V and propidium iodide (PI) according to the manufacturer's protocol (Annexin V‐FITC Apoptosis Detection Kit I, BD Pharmingen) and subsequently analyzed on a FACSCalibur flow cytometer (BD, Franklin Lakes, NJ, USA).

### 2.6 Determination of mitochondrial integrity

Eosinophils were incubated with the potential‐dependent JC‐1 dye (5 μg/ml) (Sigma–Aldrich) for 20 min at 37°C. The percentage of cells with depolarized mitochondrial membrane potential was determined by the loss of red fluorescence. Carbonyl cyanide m‐chlorophenyl hydrazon (CCCP) destroys the mitochondrial membrane potential by uncoupling oxidative phosphorylation in mitochondria and hence increasing membrane permeability to ions.

MitoTracker Red CMXRos dye (75 nM) (Life Technologies, Carlsbad, CA, USA) was added to the cell suspension for 45 min and mitochondrial membrane integrity was detected by fluorescence microscopy (Zeiss Axiovert 40 CFL microscope, Olympus DP50‐CU digital camera and Olympus CellˆP software (Olympus, Lake Success, NY, USA).

### 2.7 Cell viability assay

HEK‐DP2+DP1, HEK‐DP1, and HEK‐DP2 cells were seeded in 96well plates at a density of 3 × 10^5^ cells/ml and grown to confluence. Subsequently, cells were starved (OptiMEM, Thermo Fisher Scientific), treated with indicated concentrations of PGD_2_ and viability was determined as previously described using the CellTiter 96^®^ AQueous One Solution Cell Proliferation Assay (Promega; Madison, WI, USA)^30^ at 24 and 48 h of PGD_2_ treatment.

### 2.8 Caspase 3/7 Glo assay

Eosinophils were seeded in 96‐well plates at a density of 0.5 × 10^5^ cells/well in serum reduced medium (RPMI, 1% FBS, 1% PenStrep), and incubated for 18 h. Caspase‐Glo 3/7 assay was performed according to the manufacturer's protocol (Promega). Relative light units were detected with a chemiluminescence plate reader (TopCount NTX, Perkin Elmer/Packard Bioscience).

#### 2.8.1 Electric cell‐substrate impedance sensing

Growth performance of HEK‐DP2+DP1, HEK‐DP1, and HEK‐DP2 was monitored using the electric cell‐substrate impedance sensing (ECIS) system (Applied Biophysics, Troy, NY, USA). Cells (3 × 10^4^ cells/well) were seeded in gelatin coated 96W1E + polycarbonate arrays equipped with gold microelectrodes and allowed to settle overnight. Medium was changed to serum free (OptiMEM) medium 2 h prior to treatment with vehicle, PGD_2_ (0.1–10 μM) or FBS (10%). Impedance was measured at 4000 Hz and monitored continuously over ≥60 h.

#### 2.8.2 Microscopy

HEK‐DP2+DP1, HEK‐DP1, and HEK‐DP2 cells were seeded in 96‐well plates at a density of 3 × 10^5^ cells/ml and grown to confluency. Subsequently, cells were starved (OptiMEM) and treated with indicated concentrations of PGD_2_. Phase contrast images were taken after 48 h of culture in serum free media on a Zeiss Axiovert 40 CFL microscope and Zeiss LD A‐Plan 20×/0.30 Ph1 lens, using a Hamamatsu ORCA‐03G digital camera. Data show representative images of 4 independent experiments.

### 2.9 Gene expression analysis

Isolated peripheral blood eosinophils were lysed in TriPure and total RNA was extracted with RNeasy Mini Kit (Qiagen, Hilden, Germany) after incubation in RPMI (1% FBS, 1% PenStrep) for 3 h in the presence of 1 μM of PGD_2_, DK‐PGD_2_, or BW245c or 10 ng/ml of IL‐5. cDNA was synthesized from isolated RNA with iScript (BioRad, Hercules, CA, USA) according to manufacturer's instructions and used as the template for quantitative real time PCR (CFX, BioRad) and the use of SYBR Green Master Mix (BioRad). Relative expression of mRNA levels was calculated by normalizing to 18 s rRNA (2^−Δct^). Data are depicted as arbitrary units of vehicle control cells. The following primer pairs were used:

CCR3 (5′‐CATTGTCCATGCTGTGTTTGC‐3′; 5′‐AGGTGACGATGCTGGTGATGA‐3′), VLA‐4; (5′‐GTCCTTGTTTAATGCTGGAGATG AT‐3′; 5′‐GCTTCTCTTCCAGCTCTAAAATCTT‐3′), bcl‐xl (5′‐GCGTA GACAAGGAGATGCAGGT‐3′; 5′‐GGTCATTCAGGTAAGTGGCCAT‐3′), bax (5′‐AAAGATGGTCACGGTCTGCC‐3′; 5′‐TCCAAGACCAGGGT GGTTG‐3′),^31^ 18S (5′‐GTGGAGCGATTTGTCTGGT‐3′; 5′‐GGACA TCTAAGGCATCACAG‐3′).

### 2.10 Flow cytometric analysis of CCR3 and VLA‐4 expression

Isolated eosinophils (5 × 10^5^/ml) were kept in RPMI (Thermo Fisher Scientific) supplemented with 1% FBS and PenStrep (Sigma–Aldrich) and stimulated with 1 μM DK‐PGD_2_ or BW245c for 18 h at 37°C. Cells were stained with mouse anti‐human CD193 (CCR3)–BV421 (BioLegend, 5E8) and mouse anti‐human CD49d (VLA‐4)‐PE (BD Biosciences, 9F10), or the respective isotype controls. Human TruStain FcX (BioLegend) was used as Fc receptor blocking solution. Data were acquired in a BD FACS Canto II.

### 2.11 Statistical analysis

Data are shown as means ± sem or as individual data values. Statistical analysis was carried out with Graph Pad Prism^®^ 7 (GraphPad Software, Inc. CA, USA). Differences between groups were tested by one‐way or two‐way ANOVA followed by Dunnett's, Tukey's, or Sidak's posttest. *P* values ≤ 0.05 were considered significant and are indicated as *P ≤ 0.05; **P ≤ 0.01; ***P ≤ 0.001; and ****P ≤ 0.0001.

## 3 RESULTS

### 3.1 DP1 — but not DP2 activation promotes survival of eosinophils

We first tested the potential pro‐survival effect of the two PGD_2_ receptors DP1 and DP2 on eosinophils under ex vivo culture conditions. Isolated peripheral blood eosinophils were cultured in media containing 1% FBS for 18 h. Concurrently, eosinophils were treated with 1 μM of PGD_2_, the selective DP1 agonist BW245c, the selective DP2 agonist DK‐PGD_2_, or IL‐5 [100 pM]. IL‐5 is well known for its pro‐survival stimulus on eosinophils.^32^

**Figure 1:**
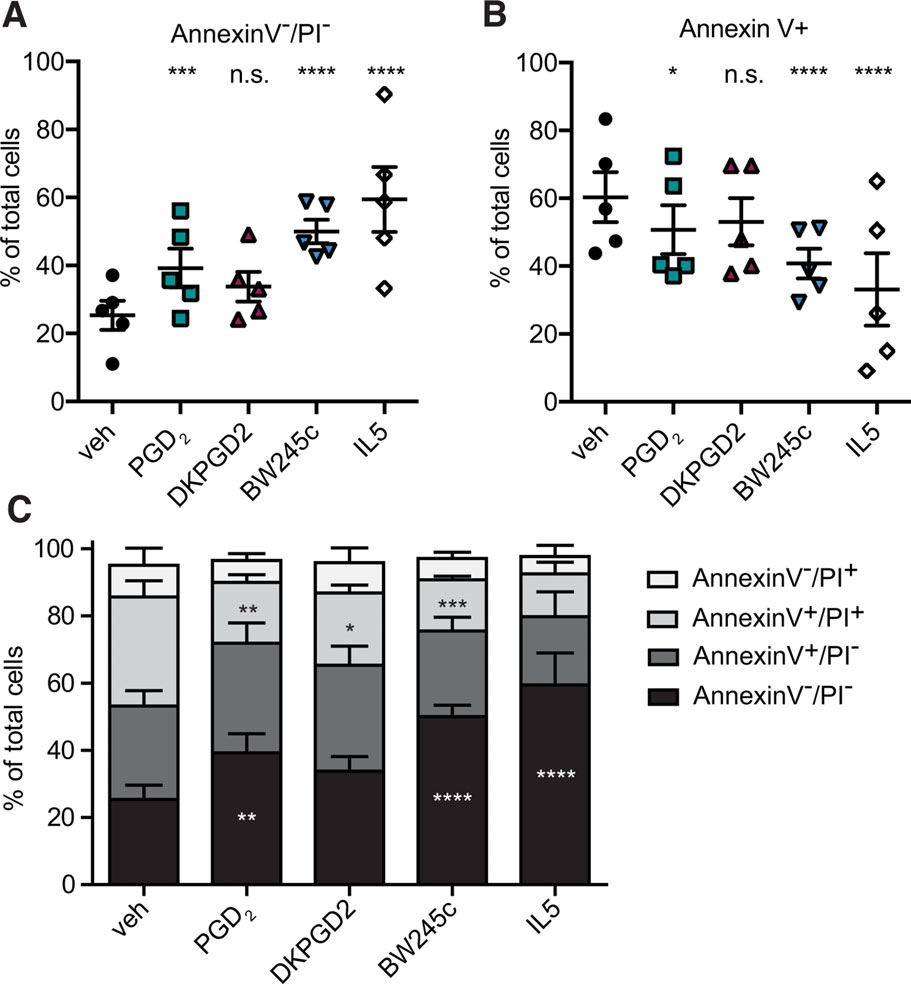
**DP1 receptor activation promotes survival of eosinophils**. Isolated eosinophils were cultured with or without 1 μM of PGD_2_, DK‐PGD_2_, BW245c, or IL‐5 [100 pM] for 18 h. BW245c, PGD_2_, and IL‐5 significantly enhanced the portion of annexin V^−^/PI^−^ eosinophils **(A)** and PGD_2_, BW245c, DK‐PGD_2_, and IL‐5 decreased the annexin V+ population **(B)** as compared to vehicle‐treated cells. **(C)** Shows the percentage of annexin V^−^/PI^−^, annexin V^+^/PI^−^, annexin V^+^/PI^+^, annexin V^−^/PI^+^ populations of total eosinophils at 18 h. Data show mean ± sem of 5 individual experiments using eosinophils from different donors

The specific DP1 agonist BW245c significantly enhanced the percentage of viable cells (Annexin V^−^/PI^−^) from 25 to 50% of all eosinophils; by comparison, IL‐5 maintained 59% of the cells viable (Fig. 1A). BW245c concentration‐dependently inhibits apoptosis of eosinophils, with a half maximal efficacy (EC50) of 0.826 μM (Supplementary Fig. 1). PGD_2_ itself moderately increased the percentage of viable cells to 39%. In contrast, the DP2 agonist DK‐PGD_2_ at the same concentration as BW245c led only to a minor enhancement of the percentage of viable cells when compared to vehicle controls.

Under the same experimental conditions, the apoptotic (Annexin V^+^) population of cultured eosinophils was reduced from 60% (vehicle control cells) to 41% (BW245c), to 51% (PGD_2_) and to 33% (IL‐5), respectively (Fig. 1B). Figure 1C discriminates between early and late apoptotic cells and depicts the distribution of single and double‐positive stained populations. Twenty‐five percent of vehicle‐treated control cells were identified as viable (Annexin V^−^/PI^−^), 28% as early apoptotic (Annexin V^+^/PI^−^), 33% were positive for both (Annexin V^+^/PI^+^), while the necrotic population (Annexin V^−^/PI^+^) represented 9% (Fig. 1C).

Of the BW245c treated eosinophils 50% were viable, 25% early‐ and 15% late‐apoptotic, and 6% were necrotic after 18 h. IL‐5 treatment led proportionally to a similar result with a slightly more pronounced effect on the increase of live cells (59% viable, 20% early‐, 13% late‐apoptotic, 8% necrotic eosinophils). Annexin V/PI staining at 0 and 3 h revealed that at these early time points there were no significant differences between these treatment groups (data not shown).

We conclude that activation of the DP1 receptor functions as a pro‐survival stimulus for eosinophils and reduces the portion of Annexin V^+^ and PI^+^ cell populations.

### 3.2 DP1 signaling enhances eosinophil survival by inhibiting the intrinsic apoptosis pathway

Since the DP1 agonist BW245c prolonged the survival of eosinophils, we aimed to identify the pro‐survival signals that were induced by DP1 receptor activation and to assess whether this function of PGD_2_ included the activation of programmed cell death pathways. Therefore, we investigated the potential involvement of DP1‐mediated signaling on the onset of the apoptotic cascade in terms of effector caspase 3/7 activation, mitochondrial membrane potential, and involvement of the anti‐apoptotic protein Bcl‐X_L_. Pore formation in the mitochondrial membrane leads to a loss of the mitochondrial membrane potential ( Δψm) and subsequently to the release of cytochrome C; inhibition of this pathway was shown to be directly linked to the recruitment and/or stabilization of anti‐apoptotic proteins of the Bcl‐2 family.^33^

**Figure 2:**
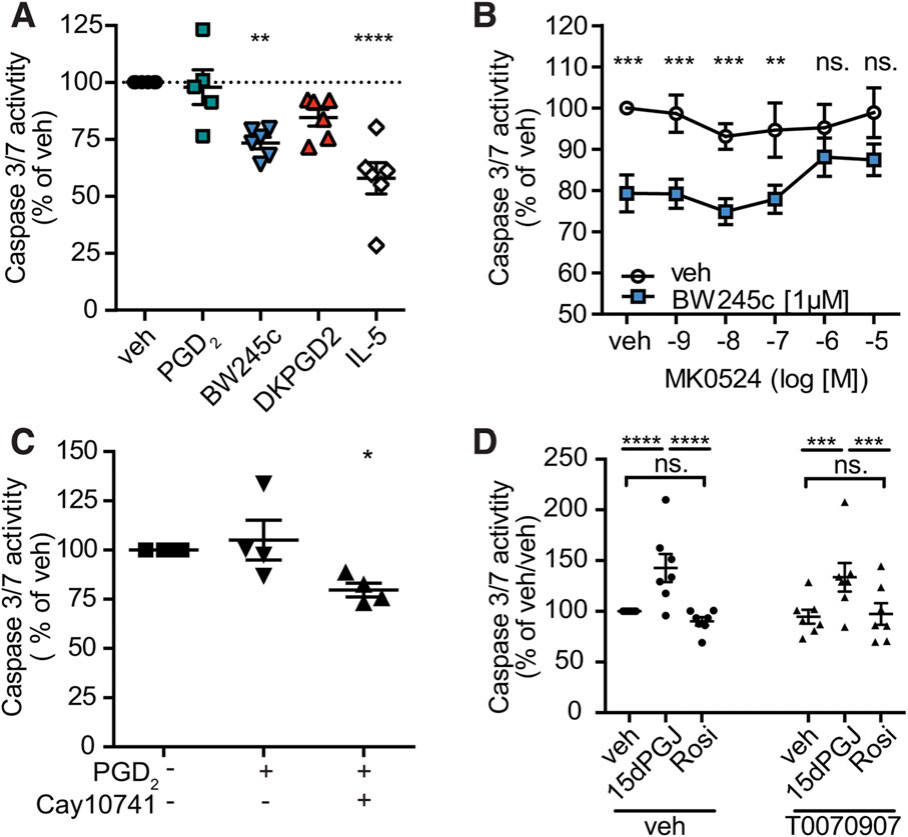
**Activation of the DP1 receptor inhibits the intrinsic apoptotic pathway in eosinophils**. Caspase 3/7 activity in eosinophils was assessed in a luminescent assay after 18 h incubation with vehicle, 1 μM of PGD_2_, BW245c, DK‐PGD_2_, or 15d‐PGJ_2_, Rosiglitazone (Rosi), or 100 pM of IL‐5. Cells were pretreated with 1 μM of vehicle, Cay10471 or T0070907 or increasing concentrations of MK0524 prior to agonist treatment. **(A)** BW245c protected from effector caspase 3/7 activity in eosinophils cultured for 18 h in serum‐reduced media (RPMI 1% FBS, 1% PenStrep). Data show means ± SEM of 5–6 individual experiments. **(B)** DP1 receptor antagonist MK0524 reversed the BW245c‐induced protection from caspase 3/7 activation. Data show mean ± sem of 9 individual experiments. **(C)** Blockade of DP2 (Cay10471) unmasks PGD_2_ as a potent inhibitor of caspase 3/7 activation. Data show mean ± sem of 4 individual experiments. **(D)** 15dPGJ_2_, but not rosiglitazone (Rosi), increased caspase 3/7 activity, which was not altered by the PPAR‐α antagonist T0070907 (*n* = 7)

BW245c significantly decreased the activity of effector caspases 3 and 7 by 27% in eosinophils aged in serum‐reduced media for 18 h, as compared to vehicle‐treated control cells (Fig. 2A). The DP1 receptor antagonist MK0524 prevented BW245c‐induced protection from caspase 3/7 activation in a concentration‐dependent manner (Fig. 2B). PGD_2_ failed to significantly decrease effector caspase activity by itself but, of note, after pharmacological blocking of DP2 receptors by the antagonist Cay10471, PGD_2_ gained the capacity to decrease caspase 3/7 activity (Fig. 2C).

PGD_2_ is a short‐lived molecule that can rapidly be metabolized to J‐metabolites such as 15‐deoxy‐Δ^12^^,^ Δ^14^‐PGJ_2_ (15dPGJ_2_), which has been reported to have a higher affinity for DP2 than DP1.^17^ 15dPGJ_2_ also activates the intracellular peroxisome proliferator‐activated receptor (PPAR)‐γ at micromolar concentrations, thereby inducing apoptosis of eosinophils.^34^ In our hands, 1 μM of 15dPGJ_2_ significantly increased caspase 3/7 activity in a PPAR‐γ‐independent manner, since 1 μM of the PPARγ T0070907 antagonist did not reverse the 15dPGJ_2_‐induced caspase 3/7 activation. Moreover, the PPAR‐γ agonist rosiglitazone (1 μM) did not mimic the 15dPGJ_2_‐induced caspase activation (Fig. 2D). T0070907 had no effect on basal caspase 3/7 activity.

### 3.3 BW245c, PGD_2_ and IL‐5 protect eosinophils from mitochondrial membrane depolarization

**Figure 3:**
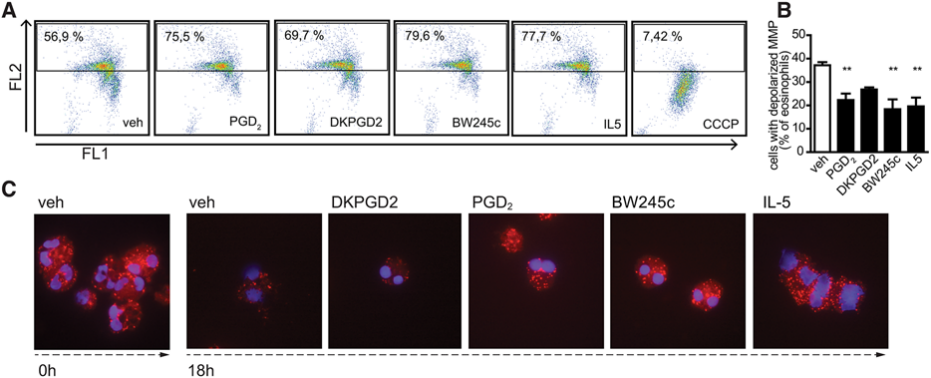
**Activation of the DP1 receptor maintains mitochondrial function in aging eosinophils. (A, B)** Flow cytometric dot plot analysis of eosinophils stained with JC1 after 18 h incubation with 1 μM of vehicle, PGD_2_, BW245c, DK‐PGD_2_, or 50 pM of IL‐5. Depolarized mitochondrial membrane potential (MMP) is shown by the loss in yellow fluorescence (FL2). **(A)** Shows representative dot plots and **(B)** means of *n* = 3–4 individual experiments ± sem. **P ≤ 0.01. **(C)** MitoTracker Red CMXRos staining of eosinophils incubated with 1 μM of vehicle, PGD_2_, BW245c, and DK‐PGD_2_, and 100 pM of IL‐5 up to 18 h. Representative images of 3 independent experiments are shown

Mitochondrial membrane depolarization is an early step of the intrinsic apoptotic cascade and can be determined by staining cells with ΔΨm‐dependent fluorescent dye JC‐1, whose fluorescent characteristics depend on the integrity of the mitochondrial membrane potential (ΔΨm). In cells with intact ΔΨm the dye binds and forms J‐aggregates at the mitochondrial membrane, and emits light at 590 nm after excitation at 527 nm. Due to the low ΔΨm in early apoptotic cells J‐aggregate formation is prevented and allows JC‐1 to be present in its monomeric form which emits light at 527 nm.^35^ Therefore, this initiating step in the onset of the intrinsic apoptotic cascade can be detected by the loss of fluorescence intensity at 590 nm. Indeed, a smaller proportion of eosinophils showed a depolarized mitochondrial membrane when cultured for 18 h in presence of PGD_2_ (22%), BW245c (18%), or IL‐5 (20%) compared to vehicle‐treated eosinophils (37%). The mitochondrial uncoupler CCCP was added to the cells for the last 30 min of culture and was used as a control for collapsed ΔΨm (Fig. 3A and B). DK‐PGD_2_ did not have a significant impact on mitochondrial membrane depolarization of eosinophils.

Additionally, eosinophils incubated with PGD_2_, BW245c, or IL‐5 showed more intense staining of intact mitochondria than vehicle‐treated cells when identified by mitochondria‐selective probes (MitoTracker Red CMXRos), which selectively accumulate and are oxidized at the mitochondrial membranes with intact membrane potential (Fig. 3C).

### 3.4 BCL‐X_L_ protein inhibitors abrogate DP1‐mediated anti‐apoptotic signaling

To investigate whether the pro‐survival effect of DP1 includes the anti‐apoptotic proteins of the Bcl‐2 family we tested the Bcl‐2 family protein inhibitors BH3I‐1 and HA14.1 with regard to their impact on apoptosis and effector caspase activation in eosinophils. Bcl‐X_L_ belongs to the family of Bcl‐2 proteins. The protein is expressed in human eosinophils and is involved in the pro‐survival signaling initiated by IL‐5 and GM‐CSF.^36^

The Bcl‐2 family inhibitor HA‐14.1 has been characterized as a ligand of a Bcl‐2 surface pocket and inhibits the binding of anti‐apoptotic Bcl‐2 proteins on the mitochondrial membrane. The small molecule compound was shown to induce apoptosis associated with mitochondrial membrane depolarization and caspase activation.^37^ Hence, we used HA14.1 to test whether Bcl‐2/Bcl‐X_L_ pathways are activated in the setting of BW245c‐mediated survival of eosinophils. A second small molecule inhibitor, BH3I‐1, acts by preventing the interaction between pro‐ and anti‐apoptotic members of the Bcl‐2 family via the BH3 domain of Bcl‐X_L_.^38^

**Figure 4:**
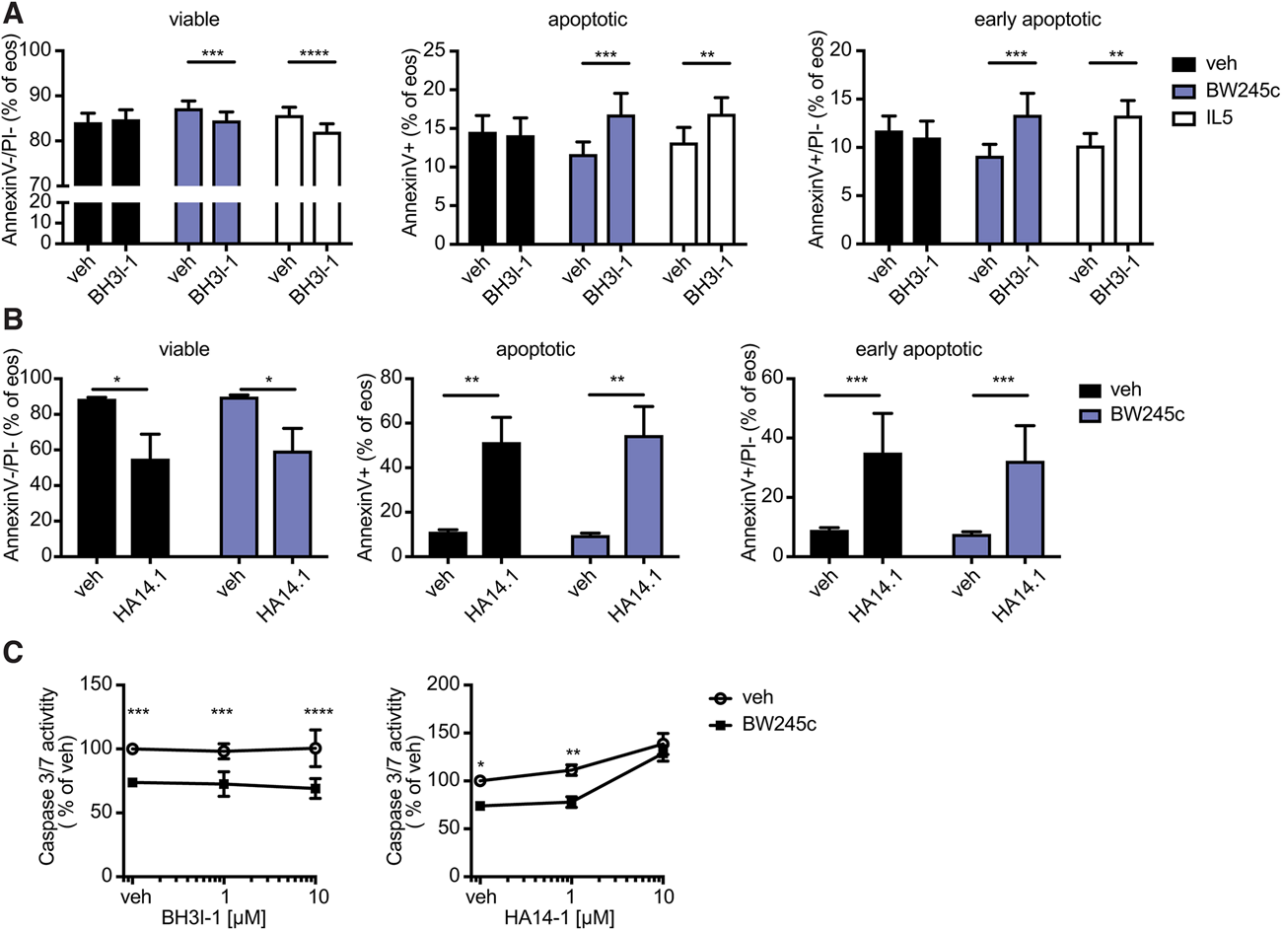
**Bcl‐2 family protein inhibitor BH3I‐1 reverses the anti‐apoptotic effect of DP1 receptor activation**. Eosinophils isolated from peripheral blood were incubated with BW245c [1 μM] in combination with or without Bcl‐X_L_ inhibitors **(A and C)** BH3l‐1 [50 μM] for 5h or **(B)** HA14.1 [10 μM] for 3 h; **(C)** 18 h. (A) BH3l‐1 prevented the reduction of annexin V^+^ apoptotic cells induced by BW245c or IL‐5. (B) HA14.1 induced apoptosis of veh‐ and BW245c‐treated eosinophils. **(C)** In contrast to BH3I‐1, HA14.1 reversed the BW245c‐induced inhibition of caspase 3/7 activity. Data show mean ± sem of 5 individual experiments

In eosinophil preparations cultured for 5 h, BH3I‐1 increased the percentage of apoptotic cells and abrogated the viability‐enhancing effects of BW245c and IL‐5 (Fig. 4A). At this early time point BW245c and IL‐5 increased the portion of annexin V‐/PI‐cells while BW245c but not IL‐5 significantly decreased the potion of annexin V+ or annexin V+/PI‐ (Supplementary Fig. 2). Similarly, HA14.1 enhanced the number of apoptotic cells in BW245c‐supplemented cultures, but it also induced apoptosis by itself already after 3 h (Fig. 4B). Moreover, 10 μM HA14.1 prevented the inhibitory effect of caspase 3/7 activity in BW235c‐treated cells (Fig. 4C) while BH3I‐1 did not interfere with BW245c‐induced caspase 3/7 inhibition at 18 h.

### 3.5 DP1 receptor expression facilitates increased viability of PGD_2_‐treated HEK293 cells

**Figure 5:**
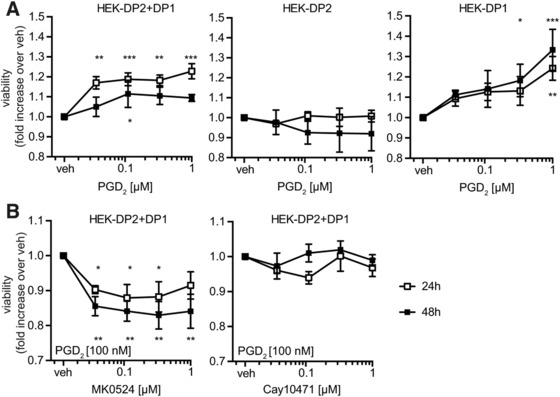
**PGD_2_ enhances the viability of HEK‐DP2+DP1 and HEK‐DP1 but not of HEK‐DP2 cells. (A)** HEK293 cell lines were starved in OptiMEM for 4 h and incubated with increasing concentrations of PGD_2_ for 24 of 48 h. Viability was detected by MTS assay (*n* = 5). **(B)** MK0524 but not Cay10471 antagonized the viability‐enhancing action of PGD_2_ on HEK‐DP2+DP1. (*n* = 3). Data show means of 3–5 independent experiments ± sem

**Figure 6:**
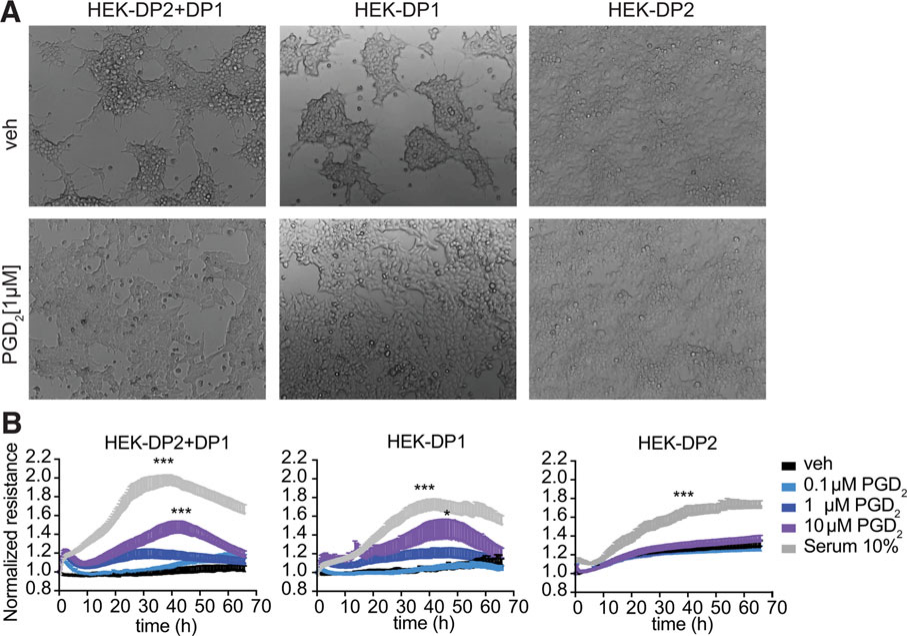
**PGD_2_ treatment causes a growth advantage of HEK‐DP2+DP1 and HEK‐DP1 but not of HEK‐DP2 cells. (A)** PGD_2_ treatment of DP1‐expressing cells led to morphologically visible growth advantage compared to HEK‐DP2 cells. Data show representative images of 4 independent experiments. **(B)** Cultures of serum‐starved HEK‐DP2+DP1, HEK‐DP1, or HEK‐DP2 cells were treated with increasing concentrations of PGD_2_ or 10% FBS and their electrical resistance was monitored for more than 60 h on an ECIS device. Data show means of 5 independent experiments + sem

Because eosinophils naturally expressed both, DP1 and DP2 receptors we used a HEK293 cell system, with stably overexpressed DP1, DP2, or both receptors in combination (HEK‐DP1, HEK‐DP2, HEK‐DP1 + DP2) and performed reporter gene assays. First, we tested whether the pro‐survival signaling of DP1 is conserved also in the HEK293 cell lines by formazan‐based viability tests (MTS). When treated with PGD_2,_ cells expressing DP1 (HEK‐DP1 and HEK‐DP1+DP2) had an advantage in maintaining viability under starving conditions (Opti‐MEM) as compared to cells lacking DP1 (HEK‐DP2) (Fig. 5A). DP1 antagonist MK0524, but not DP2 antagonist Cay10471, reversed the observed pro‐survival stimulus provided by 100 nM PGD_2_ (Fig. 5B). Further, DP1 expressing cells showed a morphologically visible advantage in growth and formation of monolayers when cultured with PGD_2_ in starving conditions as compared to HEK‐DP2 cells (Fig. 6A).

Next, we assessed the growth of HEK293 monolayers expressing DP1 and/or DP2 that were treated with PGD_2_ (0.1–10 μM), or 10% FBS as positive control stimulus. PGD_2_ increased the growth rate of HEK‐DP1 and HEK‐DP2+DP1 but not HEK‐DP2 at day 2 (Fig. 6B) as assessed by ECIS. The increase in resistance has been shown to directly correlate with the growth rate of adherent cells.^39^ Thus, besides promoting viability, in cells capable of proliferating DP1 receptor expression leads to increased growth in response to PGD_2_. Although PGD_2_ did not change the resistance of HEK‐DP2, we observed an increased basal resistance of HEK‐DP2 when compared to HEK‐DP1 or HEK‐DP2+DP1. This was probably consistent with faster proliferation of this cell line which we also observed during cell culture handling (Supplementary Fig. 4).

### 3.6 DP1 but not DP2 is a transcriptional regulator and induces serum response element

Programmed cell death can be prevented by the induction of anti‐apoptotic genes, which counteract the initiation of the apoptotic cascade. To further elucidate the pro‐survival signaling of the DP1 receptor and test the hypothesis that DP1 signaling modulates eosinophil function on a transcriptional level, we studied the potential of DP1 and DP2 to induce activation of serum response element (SRE). SRE induces the expression of anti‐apoptotic genes in the Bcl‐2 family^40^ which could further promote allergic inflammation due to prolonged survival of eosinophils. Moreover, SRE and its activator, serum response factor (SRF) might be particularly important in eosinophil migration and adhesion as SRE is a transcriptional regulator in the promotor region of genes encoding for proteins regulating the cytoskeleton such as c‐Fos or profillin.^41^ SRE was shown to regulate migration of other innate immune cells such as neutrophils^42^ and macrophages^43^ but its function in eosinophils has not been investigated yet.

**Figure 7:**
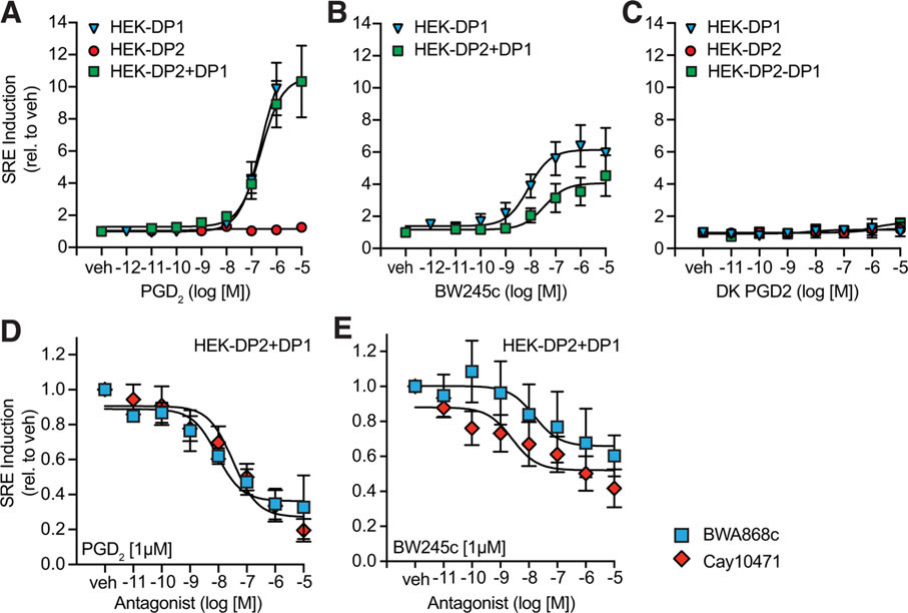
**DP1 induces SRE activation. (A)** PGD_2_ activates SRE in HEK‐DP2 + DP1 and HEK‐DP1 but not in HEK‐DP2. **(B)** DP1 agonist BW245c induces SRE activation in HEK‐DP2 + DP1 and HEK‐DP1. **(C)** DP2 agonist DK‐PGD_2_ does not induce SRE activation. **(D)** Antagonists of DP1 (BWA868c) and DP2 (Cay10471) block PGD_2_‐ and **(E)** BW245c induced SRE activation in HEK‐DP2 + DP1. Data are shown as mean ± sem of 3–6 independent experiments

In a reporter gene assay, DP1 but not DP2 was capable of inducing SRE activation in HEK‐DP1 and HEK‐DP2+DP1 cells (Fig. 7A). In contrast, no SRE activation was observed in HEK‐DP2 cells. Remarkably, however, the potency of BW245c to activate SRE was reduced when DP2 was co‐expressed (Fig. 7B). Although the DP2 agonist DK‐PGD_2_ was unable to induce SRE in HEK‐DP1+DP2 cells (Fig. 7C), blocking DP2 with the specific antagonist Cay10471 led to reduced potency of PGD_2_ and BW245c to induce SRE (Fig. 7D–E). The respective EC_50_ and IC_50_ values are shown in Supplementary Table 1. This finding highlights the crosstalk of DP1 and DP2 in a jointly regulated receptor signaling unit and suggests a modulator role of DP2 on DP1 signaling.

### 3.7 Gene expression in eosinophils is regulated by DP1

**Figure 8:**
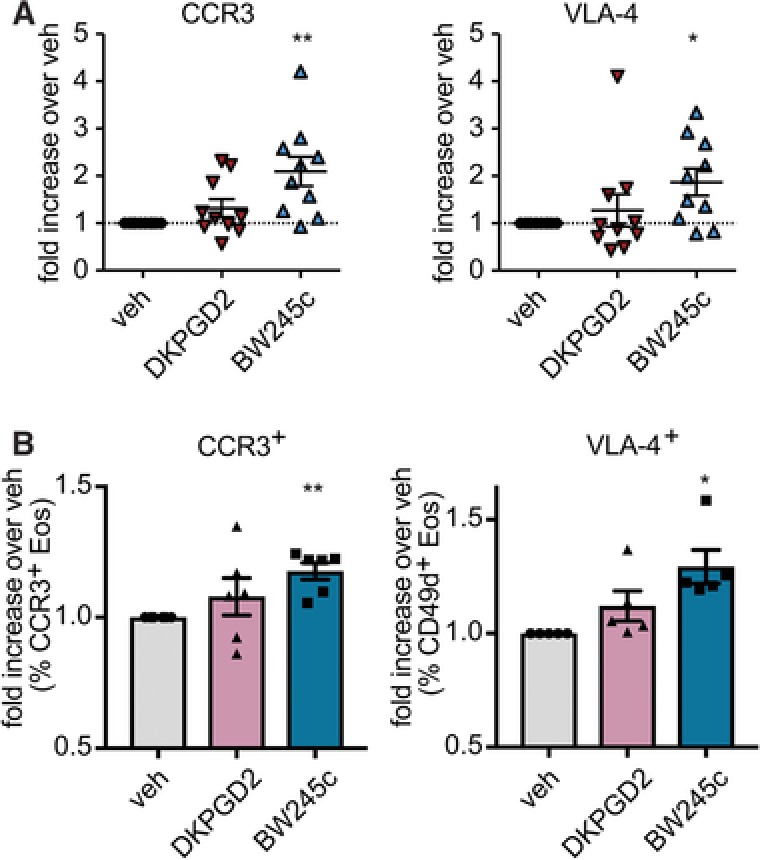
**DP1 receptor agonist BW245c enhances (A) mRNA expression of CCR3 and VLA‐4 and (B) upregulates surface expression of CCR3 and VLA‐4**. Isolated eosinophils (5 × 10^6^/ml) were incubated with vehicle (EtOH), DK‐PGD_2_ [1 μM], or BW245c [1 μM] for 3 h (A) or 18h (B) (RPMI, 1% FBS, 1%, PenStrep, 37°C). mRNA expression was measures by qRT PCR (A). Surface expression was determined by flow cytometric analysis (B). Data show mean ± sem (*n* = 5–10)

The transcription factor SRF regulates actin cytoskeleton remodeling, cell contact maintenance and adhesion^44^, ^45^, ^46^ and was shown to promote the expression of anti‐apoptotic Bcl‐X_L_ (B‐cell lymphoma‐extra‐large) and Bcl‐2 (B‐cell lymphoma 2).^46^ Thus, we tested whether DP1‐mediated signaling can affect eosinophil expression of Bcl‐X_L_ and the major chemokine receptor, CCR3, and adhesion molecule, VLA‐4. Eosinophils were treated with PGD_2_ receptor agonists or IL‐5 10 ng/ml for 3 h (RPMI 1% FBS, 37°C, 5% CO_2_), total RNA was extracted from cell lysates and mRNA expression was analyzed by qRT‐PCR. Indeed, BW245c significantly increased both, mRNA levels and protein surface expression of VLA‐4 and CCR3 in human eosinophils, while DK‐PGD_2_ was ineffective in altering the levels of expression (Fig. 8A and B). Similar, BW245c increased mRNA levels of Bcl‐X_L_ but did not enhance the level of protein expression significantly as shown by intracellular flow cytometry staining (Supplementary Fig. 5A and B).

The ratio of pro‐apoptotic BAX to anti‐apoptotic Bcl‐2 or Bcl‐X_L_ reflects the pro‐survival or pro‐apoptotic status of a cell and can predict whether programmed cell death has been initiated or not. A ratio of BCL‐2 to BAX above 1 was shown to correlate with caspase 3 activation whereas a low value (<1) has been described for cells where the onset of apoptosis is suppressed, e.g., in cancer cells.^47^ Here, IL‐5 significantly decreased the BAX/Bcl‐X_L_ ratio in eosinophils whereas BW245c moderately lowered the mean ratio to a value below 1 (Supplementary Fig. 6).

Thus, we conclude that activation of DP1 contributes to a pro‐inflammatory status by enhancing VLA‐4 and CCR3 expression and delays the onset of apoptosis in eosinophils by enhancing Bcl‐X_L_ expression.

## 4 DISCUSSION

In the present study, we report a mechanism through which the PGD_2_ receptor DP1 leads to a prolonged survival of human eosinophils. In detail, we show that the DP1 agonist BW245c suppresses the activation of effector caspases in eosinophils, and protects mitochondrial membranes from depolarization which consequently sustains viability of eosinophils under minimal culture conditions. In a recombinant cell line, DP1 induces the activation of SRE. In human eosinophils, mRNA level of Bcl‐X_L_, and the expression of CCR3 and VLA‐4 is induced by DP1 activation. Thus, the DP1 receptor might not only promote the survival and, hence, persistence of eosinophils at sites of inflammation, but also modulate the course of allergic reactions by upregulating pro‐inflammatory genes, such as VLA‐4 and CCR3 or the anti‐apoptotic gene Bcl‐X_L_. Both, VLA‐4^38^ and CCR3 are crucial in adhesion and migration, and CCR3 being the major receptor chemoattractant receptor (binding to the ligands CCL11, CCL24, CCL26, CCL7, CCL13, CCLl5, and CCL5) on eosinophils also positively regulates their survival.^49^

PGD_2_ has been shown to regulate apoptosis in a cell type specific manner. It protects Th2 cells from cytokine deprivation‐induced apoptosis via DP2 signaling,^50^ and antagonizes PGE_2_‐mediated Bax activation and hence inhibits the induction of apoptosis in glioma cells.^51^, ^52^ In contrast, PGD_2_ induces apoptosis in human osteoclasts,^53^ leukemia cells^54^ and non‐small cell lung carcinoma cell lines.^55^, ^56^ In eosinophils, PGD_2_ has been suggested to decrease the portion of late apoptotic eosinophils at nanomolar concentrations,^21^ but to induce apoptosis in micromolar concentrations (≥10 μM).^26^

In the current study, we show that DP1 prolongs the survival of eosinophils by protecting the mitochondria from the onset of intrinsic apoptosis. The DP1 agonist BW245c reduced mitochondrial membrane depolarization and decreased caspase 3/7 activation while pharmacological blockade with the DP1 antagonist reversed the inhibitory action on caspase 3/7. In general, apoptosis of eosinophils can be induced intrinsically, or extrinsically mediated via Fas receptor.^57^, ^58^ The mechanism of DP1 induced survival is consistent with the mechanism of eosinophil‐specific survival stimuli, such as IL‐5,^32^ that has been shown to inhibit the intrinsic apoptosis pathway by activating anti‐apoptotic proteins of the Bcl2 family.^59^ Additionally, DP1 receptor expression promoted viability and increased the growth of PGD_2_‐treated HEK293 monolayers. Because this finding shows that DP1 has a growth stimulating role on proliferating cells, DP1‐signaling might contribute to the differentiation and growth of eosinophil progenitor cells in addition to IL‐5.^60^ Future experiments might therefore address the role of PGD_2_ in the differentiation and proliferation of eosinophil progenitors.

Furthermore, we observed that DP1 signaling induces SRE, which in turn regulates the expression of genes that are crucial for the formation of the cytoskeleton and survival. Together with ternary complex factors, serum response factors (SRF) bind and activate SRE in the promotor of immediate early genes. Besides being crucial for the cytoskeleton integrity by regulating profilin expression, SRE activation drives anti‐apoptotic proteins, such as MCL1 or Bcl‐2.^40^, ^41^, ^46^ In B‐cells SRF is inactivated by cleavage of effector caspases in order to facilitate apoptosis pathways.^61^ Interestingly, the DP2 antagonist could block DP1‐mediated gene transcription but a DP2 agonist did not activate it by itself. This is consistent with our previous finding that the signaling of DP1 and DP2 is interlinked and can be different when DP1 and DP2 are expressed alone or in combination.^18^

The mechanism behind PGD_2_‐regulated gene transcription has remained largely unknown and it is not clear which particular DP1‐ or DP2‐dependent pathway leads to the observed SRE induction and at which level of the cascade the interaction between DP1 and DP2 may happen. DP1 has been shown to signal via activation of Gαs thereby activating adenylate cyclase and raise cAMP levels. However, we previously demonstrated that DP1 cannot only couple to Gαs‐,^62^, ^63^ but also to the Gαq^18^ subunit of heteromeric G‐proteins. Moreover, the Ca^2+^ signaling of DP2 is mediated by Gαi coupling, which significantly loses potency when Gαq is inhibited and DP1 co‐expressed.^18^ DP2 signaling can also be regulated by β arrestins^64^ but the signaling cascades that are directly activated by the Gβγ subunit linked to DP1 or DP2 have been barely investigated yet.

Moreover, we report that the DP1 agonist induced Bcl‐X_L_ mRNA upregulation and pharmacological inhibition of Bcl‐X_L_ reversed its pro‐survival effect. It is known that Bcl‐X_L_ is expressed in eosinophils and contributes to the pro‐survival function of IL‐5 and GM‐CSF,^36^, ^59^ and is transcriptionally regulated by SRF.^46^ Bcl‐X_L_ mRNA expression is upregulated in eosinophils from atopic dermatitis patients in comparison to eosinophils from healthy controls.^31^ Therefore, our finding supports the significance of Bcl‐X_L_ in enhanced eosinophils survival.

The BcL‐X_L_ inhibitor HA14.1 is a strong inducer of apoptosis, and although HA14.1 completely reversed the pro‐survival effect of BW245c, we cannot rule out an unspecific effect of HA14.1 since the inhibitor induced apoptosis in all samples regardless of treatment. Hence, it is difficult to decide whether HA14.1 inhibits the signaling cascades activated by BW245c. On the other hand, BH3I‐1 specifically reversed the increase of viable cells by BW245c treatment to levels of vehicle‐treated cells. Moreover, both BH3I‐1 and HA14.1 abrogated DP1‐mediated caspase 3/7 inhibition. A total of 10 μM HA14.1 induced effector caspase activation by itself which, at this concentration, could have potentially masked BW245c signaling at this concentration.

Polymorphisms, methylation, and expression patterns of PTGDR, the gene encoding the DP1 receptor, have been associated with asthma.^65^, ^66^ Therefore, altered DP1 receptor signaling might be crucial in allergic inflammation. Indeed, we could show that the DP1 receptor agonist upregulated CCR3 and VLA‐4 mRNA expression. Besides inducing chemotaxis toward eotaxin, CCR3 was shown to enhance eosinophil viability^49^ and to be transcriptionally upregulated in allergic asthma.^67^ Eosinophils bind to endothelial cells mainly via VLA‐4–VCAM interaction^68^ which—along with CCR3 activation—might be a critical regulator of eosinophil extravasation.

Enhanced survival of immune cells at inflammatory sites has been observed in the following allergic conditions^6^: (i) peripheral blood eosinophils from asthmatic patients survive longer compared to eosinophils from healthy control subjects,^69^ (ii) apoptosis rate of sputum eosinophils correlates with exhaled nitric oxide in asthmatic children,^70^ and (iii) delayed apoptosis contributes to tissue eosinophilia in nasal polyps.^71^ Moreover, there is substantial literature on the beneficial effects of the reduction of circulating eosinophils in eosinophilic asthma.^7^ In fact, depletion of eosinophils by antibody‐dependent cell‐mediated cytotoxicity (ADCC) has been recently shown to effectively improve airway resistance and disease scores in asthma patients with ≥300 eosinophils/μl blood.^72^, ^73^ The anti‐IL‐5Rα antibody benralizumab (MEDI‐563) binds to circulating eosinophils and induces apoptosis and ADCC of eosinophils and basophils in vitro. The compound depletes human bone marrow derived mononuclear cells of eosinophil precursors and eosinophils of peripheral blood of nonhuman primates and binds to eosinophils in nonhuman primate lung tissue (bronchi, small airways, bronchial parenchyma).^74^

In the present study, we observed that PGD_2_ did not decrease effector caspase activity and only moderately enhanced eosinophil viability compared to the DP1 agonist, but gained in anti‐apoptotic capacity when DP2 signaling was blocked by the respective antagonist. Furthermore, we confirm that the DP2 selective PGD_2_ metabolite 15dPGJ_2_ induces caspase activity in eosinophils in a PPAR‐ɣ independent manner.^26^ 15dPGJ_2_ was shown to induce apoptosis in cardiomyocytes via activation of DP2/MAPK/TNF‐α^75^ and via ROS formation in non‐small lung cancer cells.^76^

Our data supports previous literature demonstrating the cooperative pro‐inflammatory signaling of DP1 and DP2. PGD_2_ induces leukotriene C4 production in eosinophils only sufficiently by simultaneous activation of DP1 and DP2^77^. In guinea pigs, the mobilization of eosinophils from the bone marrow can be stimulated by both, DP1 and DP2^25^. We have shown previously that DP1 receptors are profoundly involved in the DP2‐triggered Ca^2+^ signaling and that DP1 and DP2 receptors are co‐localized and form heteromeric units in HEK‐DP2 + DP1 cells.^18^

In contrast to cooperative pro‐inflammatory functions of DP1/DP2, there is evidence that DP1 receptor counteracts CD11b upregulation in human eosinophils,^22^ and, in mice, DP1 diminishes allergic inflammation in OVA models of experimental asthma as shown by decreased lung‐eosinophilia and airway hyperresponsiveness after intratracheal administration of BW245c.^78^, ^79^ However, in mice, DP1 activation was also shown to promote allergic inflammation since DP1‐deficient mice have reduced number of eosinophils and cytokine levels (IL‐4, IL‐5, IL‐13) in the BAL fluid in OVA models of experimental asthma.^13^ Hence, we tested if PGD_2_ receptors directly regulate survival of ex vivo differentiated murine bone marrow‐derived eosinophils (bmEos). We found that neither PGD_2_ nor BW245c or DK‐PGD_2_ significantly changed the in vitro survival of bmEos significantly (Supplementary Fig. 7). Therefore, the reported roles of DP1 in vivo could result from indirect effects mediated through other cell types such as lung dendritic cells and regulatory T‐cells^71^ and it is likely that the functions of DP1 differs between human and murine eosinophils.

For the development of future therapies based on DP2 antagonists, it is hence important to consider that such compounds could potentially enhance the survival of eosinophils by shunting the signaling cascade towards anti‐apoptotic DP1‐dependent pathways. Since targeting the trafficking and, hence, the infiltration of eosinophils to the sites of inflammation seems insufficient, additionally targeting the survival of circulating and tissue‐resident eosinophils could be accomplished with dual DP1/DP2 antagonists which should be addressed in future studies.

Targeting eosinophil survival by DP1 receptor antagonists may interfere with recently discovered homeostatic functions of eosinophils. Eosinophils modulate the functions of several other immune cell types, such as T cells, B cells, mast cells, macrophages, and neutrophils, to promote a Th2‐type of inflammation^80^ but they have also been shown to directly exert antiviral and antimicrobial host defense mechanisms and modulate the functions of lymphocytes. Thereby eosinophils support the resolution of inflammatory conditions.^81^ In addition, a population of homeostatic eosinophils (hEos) that differs phenotypically and functionally from inflammatory eosinophils (iEos) has been discovered recently. hEos in the lung express genes that negatively regulate Th2 cell functions to maintain lung immune homeostasis.^82^ These findings might be important to consider in a potential therapy where DP1 receptors are targeted to prevent eosinophil survival in tissues.

On the other hand, no negative side effects of eosinophil depletion by benralizumab^74^ have been reported so far. Benralizumab is generally well tolerated and effective in patients with severe eosinophilic asthma.^83^

Interestingly, in a recent proteome analysis DP2, cyclooxygenase‐1, and prostaglandin E2 synthase, but neither DP1 nor EP2, EP4, or IP receptors were detected in peripheral blood eosinophils of allergic donors.^84^ This finding confirms the low expression level of these GPCRs which we found in eosinophils from healthy donors by Western blot and flow cytometry.^85^, ^86^, ^87^

In conclusion, our data demonstrate that the PGD_2_ receptor DP1 has a critical role in regulating the survival of eosinophils by inhibiting the onset of the intrinsic apoptotic cascade.

Hence, PGD_2_ has a dual role in allergic inflammation, that is, besides directly recruiting eosinophils to afflicted tissues, this lipid mediator might also counteract the resolution of the allergic response by prolonging the survival of eosinophils. Consequently, DP1 receptor antagonists—in addition to DP2 antagonists—or dual DP1/DP2 antagonists might be useful therapeutic tools to reduce eosinophils infiltration and activation at sites of allergic inflammation.

## Supporting information

supplementary information

## References

[1] Modena BD , Dazy K , White AA . Emerging concepts: mast cell involvement in allergic diseases. Transl Res. 2016;174:98–121. 26976119 10.1016/j.trsl.2016.02.011

[2] Kagalwalla AF , Akhtar N , Woodruff SA , et al. Eosinophilic esophagitis: epithelial mesenchymal transition contributes to esophageal remodeling and reverses with treatment. J Allergy Clin Immunol. 2012;129:1387–1396. e7. 22465212 10.1016/j.jaci.2012.03.005 PMC3340537

[3] Yasukawa A , Hosoki K , Toda M , et al. Eosinophils promote epithelial to mesenchymal transition of bronchial epithelial cells. PLoS One. 2013;8:e64281. 23700468 10.1371/journal.pone.0064281 PMC3660301

[4] Aceves SS , Broide DH . Airway fibrosis and angiogenesis due to eosinophil trafficking in chronic asthma. Curr Mol Med. 2008;8:350–358. 18691061 10.2174/156652408785161023

[5] Wilson SJ , Rigden HM , A WardJ , et al. The relationship between eosinophilia and airway remodelling in mild asthma. Cea. 2013;43:1342–1350. 10.1111/cea.12156 PMC4040270 24261944

[6] Ohta K , Yamashita N . Apoptosis of eosinophils and lymphocytes in allergic inflammation. J Allergy Clin Immunol. 1999;104:14–21. 10400833 10.1016/s0091-6749(99)70107-7

[7] Ortega HG , Liu MC , Pavord ID , et al. Mepolizumab treatment in patients with severe eosinophilic asthma. N Engl J Med. 2014;371:1198–1207. 25199059 10.1056/NEJMoa1403290

[8] Flood‐Page PT , Menzies‐Gow AN , Kay AB , Robinson DS . Eosinophil's role remains uncertain as anti–interleukin‐5 only partially depletes numbers in asthmatic airway. Am J Respir Crit Care Med. 2003;167:199–204. 12406833 10.1164/rccm.200208-789OC

[9] Castro M , Mathur S , Hargreave F , et al. Reslizumab for poorly controlled, eosinophilic asthma. Am J Respir Crit Care Med. 2011;184:1125–1132. 21852542 10.1164/rccm.201103-0396OC

[10] Naclerio RM , Meier HL , Kagey‐Sobotka A , et al. Mediator release after nasal airway challenge with allergen. Am Rev Respir Dis. 1983;128:597–602. 6354022 10.1164/arrd.1983.128.4.597

[11] Lewis RA , Soter NA , Diamond PT , et al. Prostaglandin D2 generation after activation of rat and human mast cells with anti‐IgE. J Immunol. 1982;129:1627–1631. 6809826

[12] Peinhaupt M , Sturm EM , Heinemann A . Prostaglandins and their receptors in eosinophil function and as therapeutic targets. Front Med. 2017;4:104. 10.3389/fmed.2017.00104 PMC5515835 28770200

[13] Matsuoka T , Hirata M , Tanaka H , et al. Prostaglandin D2 as a mediator of allergic asthma. Science. 2000;287:2013–2017. 10720327 10.1126/science.287.5460.2013

[14] Shen Z‐J , Esnault S , Schinzel A , Borner C , Malter JS . The peptidyl‐prolyl isomerase Pin1 facilitates cytokine‐induced survival of eosinophils by suppressing Bax activation. Nat Immunol. 2009;10:257–265. 19182807 10.1038/ni.1697 PMC2847832

[15] Pienkowski MM , Adkinson NF , Plaut M , Norman PS , Lichtenstein LM . Prostaglandin D2 and histamine during the immediate and the late‐phase components of allergic cutaneous responses. J Allergy Clin Immunol. 1988;82:95–100. 3292634 10.1016/0091-6749(88)90057-7

[16] Zhang S , Wu X , Yu S . Prostaglandin D2 receptor d‐type prostanoid receptor 2 mediates eosinophil trafficking into the esophagus. Dis Esophagus. 2014;27:601–606. 24165271 10.1111/dote.12118 PMC4000277

[17] Sawyer N , Cauchon E , Chateauneuf A , et al. Molecular pharmacology of the human prostaglandin D2 receptor, CRTH2. Br J Pharmacol. 2002;137:1163–1172. 12466225 10.1038/sj.bjp.0704973 PMC1573602

[18] Sedej M , Schröder R , Bell K , et al. D‐type prostanoid receptor enhances the signaling of chemoattractant receptor‐homologous molecule expressed on T H2 cells. J Allergy Clin Immunol. 2012;129:492–500. e9. 21930295 10.1016/j.jaci.2011.08.015

[19] Nagata K , Hirai H . The second PGD2 receptor CRTH2: structure, properties, and functions in leukocytes. Prostaglandins, Leukot Essent Fatty Acids. 2003;69:169–177. 12895600 10.1016/s0952-3278(03)00078-4

[20] Hirai H , Tanaka K , Yoshie O , et al. Prostaglandin D2 selectively induces chemotaxis in T helper type 2 cells, eosinophils, and basophils via seven‐transmembrane receptor CRTH2. J Exp Med. 2001;193:255–261. 11208866 10.1084/jem.193.2.255 PMC2193345

[21] Gervais FG , Cruz RP , Chateauneuf A , et al. Selective modulation of chemokinesis, degranulation, and apoptosis in eosinophils through the PGD2 receptors CRTH2 and DP. J Allergy Clin Immunol. 2001;108:982–988. 11742277 10.1067/mai.2001.119919

[22] Monneret G , Gravel S , Diamond M , Rokach J , Powell WS . Prostaglandin D2 is a potent chemoattractant for human eosinophils that acts via a novel DP receptor. Blood. 2001;98:1942–1948. 11535533 10.1182/blood.v98.6.1942

[23] Heinemann A , Schuligoi R , Sabroe I , Hartnell A , Peskar BA . Delta 12‐prostaglandin J2, a plasma metabolite of prostaglandin D2, causes eosinophil mobilization from the bone marrow and primes eosinophils for chemotaxis. J Immunol. 2003;170:4752–4758. 12707356 10.4049/jimmunol.170.9.4752

[24] Royer JF , Schratl P , Lorenz S , et al. A novel antagonist of CRTH2 blocks eosinophil release from bone marrow, chemotaxis and respiratory burst. Allergy Eur J Allergy Clin Immunol. 2007;62:1401–1409. 10.1111/j.1398-9995.2007.01452.x 17714552

[25] Schratl P , Royer JF , Kostenis E , et al. The role of the prostaglandin D2 receptor, DP, in eosinophil trafficking. J Immunol. 2007;179:4792–4799. 17878378 10.4049/jimmunol.179.7.4792

[26] Ward C , Dransfield I , Murray J , et al. Prostaglandin D2 and its metabolites induce caspase‐dependent granulocyte apoptosis that is mediated via inhibition of IκBα degradation using a peroxisome proliferator‐activated receptor‐γ‐independent mechanism. J Immunol. 2002;168:6232–6243. 12055237 10.4049/jimmunol.168.12.6232

[27] Hartnell A , Heinemann A , Conroy DM , et al. Identification of selective basophil chemoattractants in human nasal polyps as insulin‐like growth factor‐1 and insulin‐like growth factor‐2. J Immunol. 2004;173:6448–6457. 15528386 10.4049/jimmunol.173.10.6448

[28] Henstridge CM , Balenga NAB , Ford LA , et al. The GPR55 ligand L‐alpha‐lysophosphatidylinositol promotes RhoA‐dependent Ca2+ signaling and NFAT activation. FASEB J. 2009;23:183–193. 18757503 10.1096/fj.08-108670

[29] Balenga NA , Martínez‐Pinilla E , Kargl J , et al. Heteromerization of GPR55 and cannabinoid CB2 receptors modulates signalling. Br J Pharmacol. 2014;171:5387–5406. 25048571 10.1111/bph.12850 PMC4294047

[30] Kargl J , Haybaeck J , Stančić A , et al. O‐1602, an atypical cannabinoid, inhibits tumor growth in colitis‐associated colon cancer through multiple mechanisms. J Mol Med. 2013;91:449–458. 22965195 10.1007/s00109-012-0957-1 PMC3529923

[31] Ogawa K , Hashida R , Miyagawa M , et al. Analysis of gene expression in peripheral blood eosinophils from patients with atopic dermatitis and in vitro cytokine‐stimulated blood eosinophils. Clin Exp Immunol. 2003;131:436–445. 12605696 10.1046/j.1365-2249.2003.02090.x PMC1808659

[32] Yamaguchi Y , Hayashi Y , Sugama Y , et al. Highly purified murine interleukin 5 (IL‐5) stimulates eosinophil function and prolongs in vitro survival. J Exp Med. 1988;167:1737–1742. 2835420 10.1084/jem.167.5.1737 PMC2188945

[33] Kluck RM , Bossy‐wetzel E , Green DR , Newmeyer DD . The release of cytochrome c from mitochondria : A primary site for bcl‐2 regulation of apoptosis. Science. 1997;1275:1132–1136. 10.1126/science.275.5303.1132 9027315

[34] Ueki S , Kato H , Kobayashi Y , et al. Anti‐ and Proinflammatory effects of 15‐Deoxy‐Δ^12,14^‐Prostaglandin J_2_(15d‐PGJ_2_) on human eosinophil functions. Int Arch Allergy Immunol. 2007;143:15–22. 17541271 10.1159/000101399

[35] Salvioli S , Ardizzoni A , Franceschi C , Cossarizza A . JC‐1, but not DiOC 6 (3) or rhodamine 123, is a reliable fluorescent probe to assess ΔΨ changes in intact cells: implications for studies on mitochondrial functionality during apoptosis. FEBS Lett. 1997;411:77–82. 9247146 10.1016/s0014-5793(97)00669-8

[36] Dibbert B , Daigle I , Braun D , et al. Role for Bcl‐xL in delayed eosinophil apoptosis mediated by granulocyte‐macrophage colony‐stimulating factor and interleukin‐5. Blood. 1998;92:778–783. 9680344

[37] Wang J‐LL , Liu D , Zhang Z‐JJ , et al. Structure‐based discovery of an organic compound that binds Bcl‐2 protein and induces apoptosis of tumor cells. Proc Natl Acad Sci USA. 2000;97:7124–7129. 10860979 10.1073/pnas.97.13.7124 PMC16510

[38] Degterev A , Lugovskoy A , Cardone M , et al. Identification of small‐molecule inhibitors of interaction between the BH3 domain and Bcl‐x L. Nat Cell Biol. 2001;3:173–182. 11175750 10.1038/35055085

[39] Zudaire E , Cuesta N , Murty V , et al. The aryl hydrocarbon receptor repressor is a putative tumor suppressor gene in multiple human cancers. J Clin Invest. 2008;118:640–650. 18172554 10.1172/JCI30024 PMC2157559

[40] Vickers ER , Kasza A , Kurnaz IA , et al. Ternary complex factor‐serum response factor complex‐regulated gene activity is required for cellular proliferation and inhibition of apoptotic cell death. Mol Cell Biol. 2004;24:10340–10351. 15542842 10.1128/MCB.24.23.10340-10351.2004 PMC529045

[41] Miano JM , Long X , Fujiwara K . Serum response factor: master regulator of the actin cytoskeleton and contractile apparatus. Am J Physiol Cell Physiol. 2007;292:C70–81. 16928770 10.1152/ajpcell.00386.2006

[42] Taylor A , Tang W , Bruscia EM , et al. SRF is required for neutrophil migration in response to inflammation. Blood. 2014;123:3027–3036. 24574460 10.1182/blood-2013-06-507582 PMC4014845

[43] Sullivan AL , Benner C , Heinz S , et al. Serum response factor utilizes distinct promoter‐ and enhancer‐based mechanisms to regulate cytoskeletal gene expression in macrophages. Mol Cell Biol. 2011;31:861–875. 21135125 10.1128/MCB.00836-10 PMC3028656

[44] Ragu C , Elain G , Mylonas E , et al. The transcription factor Srf regulates hematopoietic stem cell adhesion. Blood. 2010;116:4464–4473. 20709909 10.1182/blood-2009-11-251587

[45] Medjkane S , Perez‐Sanchez C , Gaggioli C , Sahai E , Treisman R . Myocardin‐related transcription factors and SRF are required for cytoskeletal dynamics and experimental metastasis. Nat Cell Biol. 2009;11:257–268. 19198601 10.1038/ncb1833 PMC6089348

[46] Schratt G , Philippar U , Hockemeyer D , et al. SRF regulates Bcl‐2 expression and promotes cell survival during murine embryonic development. EMBO J. 2004;23:1834–1844. 15057274 10.1038/sj.emboj.7600188 PMC394242

[47] Liu F‐T , Goff LK , Hao J‐H , Newland AC , Jia L . Increase in the ratio of mitochondrial Bax/Bcl‐XL induces Bax activation in human leukemic K562 cell line. Apoptosis. 2004;9:377–384. 15258470 10.1023/b:appt.0000025815.78761.5c

[48] Das AM , Williams TJ . Lobb R , Nourshargh S . Lung eosinophilia is dependent on IL‐5 and the adhesion molecules CD18 and VLA‐4, in a guinea‐pig model. Immunology. 1995;84:41–46. 7534262 PMC1415177

[49] Shinagawa K , Trifilieff A , Anderson GP . Involvement of CCR3‐reactive chemokines in eosinophil survival. Int Arch Allergy Immunol. 2003;130:150–157. 12673069 10.1159/000069005

[50] Xue L , Fergusson J , Salimi M , et al. Prostaglandin D2 and leukotriene E4 synergize to stimulate diverse TH2 functions and TH2 cell/neutrophil crosstalk. J Allergy Clin Immunol. 2015;135:1358–1366. 25441644 10.1016/j.jaci.2014.09.006 PMC4418751

[51] Lalier L , Cartron P‐F , Pedelaborde F , et al. Increase in PGE2 biosynthesis induces a Bax dependent apoptosis correlated to patients’ survival in glioblastoma multiforme. Oncogene. 2007;26:4999–5009. 17369862 10.1038/sj.onc.1210303

[52] Lalier L , Cartron P‐F , Olivier C , et al. Prostaglandins antagonistically control Bax activation during apoptosis. Cell Death Differ. 2011;18:528–537. 20966963 10.1038/cdd.2010.128 PMC3131998

[53] Yue L , Haroun S , Parent J‐L , de Brum‐Fernandes AJ . Prostaglandin D2 induces apoptosis of human osteoclasts through ERK1/2 and Akt signaling pathways. Bone. 2014;60:112–121. 24345643 10.1016/j.bone.2013.12.011

[54] Chen Y‐C , Shen S‐C , Tsai S‐H . Prostaglandin D2 and J2 induce apoptosis in human leukemia cells via activation of the caspase 3 cascade and production of reactive oxygen species. Biochim Biophys Acta. 2005;1743:291–304. 15843042 10.1016/j.bbamcr.2004.10.016

[55] Wang J , Mak O . Induction of apoptosis in non‐small cell lung carcinoma A549 cells by PGD2 metabolite, 15d‐PGJ 2. Cell Biol Int. 2011;35:1089–1096. 21999315 10.1042/CBI20110707

[56] Ramer R , Heinemann K , Merkord J , et al. COX‐2 and PPAR‐g confer cannabidiol‐induced apoptosis of human lung cancer cells. Mol Cancer Ther. 2013;12:69–82. 23220503 10.1158/1535-7163.MCT-12-0335

[57] Segal M , Niazi S , Simons MP , Galati SA , Zangrilli JG . Bid activation during induction of extrinsic and intrinsic apoptosis in eosinophils. Immunol Cell Biol. 2007;85:518–524. 17549073 10.1038/sj.icb.7100075

[58] Tsuyuki S , Bertrand C , Erard F , et al. Activation of the Fas receptor on lung eosinophils leads to apoptosis and the resolution of eosinophilic inflammation of the airways. J Clin Invest. 1995;96:2924–2931. 8675664 10.1172/JCI118364 PMC186004

[59] Schwartz C , Willebrand R , Huber S , et al. Eosinophil‐specific deletion of IkBa in mice reveals a critical role of NF‐kB–induced Bcl‐xL for inhibition of apoptosis. Blood. 2015;125:3896–3905. 25862560 10.1182/blood-2014-10-607788 PMC4473117

[60] Clutterbuck EJ , Sanderson CJ . Human Eosinophil Hematopoiesis studied in vitro by means of murine eosinophil differentiation factor (1l5): production of functionally active eosinophils from normal human bone marrow. Blood. 1988;71:646–651. 3257886

[61] Drewett V , Devitt A , Saxton J , et al. Serum response factor cleavage by caspases 3 and 7 linked to apoptosis in human bjab cells. J Biol Chem. 2001;276:33444–33451. 11387340 10.1074/jbc.M103877200

[62] Hata AN , Breyer RM . Pharmacology and signaling of prostaglandin receptors: multiple roles in inflammation and immune modulation. Pharmacol. Ther. 2004;103:147–166. 15369681 10.1016/j.pharmthera.2004.06.003

[63] Hirata M , A Kakizuk , Aizawa M , Ushikubi F , Narumiya S . Molecular characterization of a mouse prostaglandin D receptor and functional expression of the cloned gene. Proc Natl Acad Sci USA. 1994;91:11192–11196. 7972033 10.1073/pnas.91.23.11192 PMC45193

[64] Schröder R , Merten N , Mathiesen JM , et al. The C‐terminal Tail of CRTH2 is a key molecular determinant that constrains gα _i_ and downstream signaling cascade activation. J Biol Chem. 2009;284:1324–1336. 19010788 10.1074/jbc.M806867200

[65] Isidoro‐García M , Sanz C , García‐Solaesa V , et al. PTGDR gene in asthma: a functional, genetic, and epigenetic study. Allergy. 2011;66:1553–1562. 21883277 10.1111/j.1398-9995.2011.02685.x

[66] Oguma T , Palmer LJ , Birben E , et al. Role of prostanoid DP receptor variants in susceptibility to asthma. N Engl J Med. 2004;351:1752–1763. 15496624 10.1056/NEJMoa031785

[67] Ying S , Robinson DS , Meng Q , et al. Enhanced expression of eotaxin and CCR3 mRNA and protein in atopic asthma. association with airway hyperresponsiveness and predominant co‐localization of eotaxin mRNA to bronchial epithelial and endothelial cells. Eur J Immunol. 1997;27:3507–3516. 9464841 10.1002/eji.1830271252

[68] Dobrina A , Menegazzi R , Carlos TM , et al. Mechanisms of eosinophil adherence to cultured vascular endothelial cells. Eosinophils bind to the cytokine‐induced ligand vascular cell adhesion molecule‐1 via the very late activation antigen‐4 integrin receptor. J Clin Invest. 1991;88:20–26. 1711540 10.1172/JCI115278 PMC295997

[69] Kankaanranta H , Lindsay MA , Giembycz MA , et al. Delayed eosinophil apoptosis in asthma. J Allergy Clin Immunol. 2000;106:77–83. 10887309 10.1067/mai.2000.107038

[70] Pontin J , Blaylock MG , Walsh GM , Turner SW . Sputum eosinophil apoptotic rate is positively correlated to exhaled nitric oxide in children. Pediatr Pulmonol. 2008;43:1130–1134. 18972415 10.1002/ppul.20921

[71] Simon HU , Yousefi S , Schranz C , et al. Direct demonstration of delayed eosinophil apoptosis as a mechanism causing tissue eosinophilia. J Immunol. 1997;158:3902–3908. 9103460

[72] FitzGerald JM , Bleecker ER , Nair P , et al. Benralizumab, an anti‐interleukin‐5 receptor α monoclonal antibody, as add‐on treatment for patients with severe, uncontrolled, eosinophilic asthma (CALIMA): a randomised, double‐blind, placebo‐controlled phase 3 trial. Lancet. 2016;388:2128–2141. 27609406 10.1016/S0140-6736(16)31322-8

[73] Ferguson GT , FitzGerald JM , Bleecker ER , et al. Benralizumab for patients with mild to moderate, persistent asthma (BISE): a randomised, double‐blind, placebo‐controlled, phase 3 trial. Lancet Respir Med. 2017;2600:1–9. 10.1016/S2213-2600(17)30190-X 28545978

[74] Kolbeck R , Kozhich A , Koike M , et al. MEDI‐563, a humanized anti‐IL‐5 receptor α mAb with enhanced antibody‐dependent cell‐mediated cytotoxicity function. J Allergy Clin Immunol. 2010;125:1344–1353. e2. 20513525 10.1016/j.jaci.2010.04.004

[75] Koyani CN , Windischhofer W , Rossmann C , et al. 15‐deoxy‐Δ^12^,^14^‐PGJ₂ promotes inflammation and apoptosis in cardiomyocytes via the DP2/MAPK/TNFα axis. Int J Cardiol. 2014;173:472–480. 24698234 10.1016/j.ijcard.2014.03.086 PMC4008937

[76] Wang J‐J , Mak O‐T . Induction of apoptosis by 15d‐PGJ2 via ROS formation: An alternative pathway without PPARγ activation in non‐small cell lung carcinoma A549 cells. Prostaglandins Other Lipid Mediat. 2011;94:104–111. 21396480 10.1016/j.prostaglandins.2011.01.004

[77] Mesquita‐Santos FP , Bakker‐Abreu I , Luna‐Gomes , et al. Co‐operative signalling through DP(1) and DP(2) prostanoid receptors is required to enhance leukotriene C(4) synthesis induced by prostaglandin D(2) in eosinophils. Br J Pharmacol. 2011;162:1674–1685. 20973774 10.1111/j.1476-5381.2010.01086.x PMC3081113

[78] Spik I , Brénuchon C , Angéli V , et al. Activation of the prostaglandin D2 receptor DP2/CRTH2 increases allergic inflammation in mouse. J Immunol. 2005;174:3703–3708. 15749909 10.4049/jimmunol.174.6.3703

[79] Hammad H , Kool M , Soullié T , et al. Activation of the D prostanoid 1 receptor suppresses asthma by modulation of lung dendritic cell function and induction of regulatory T cells. J Exp Med. 2007;204:357–367. 17283205 10.1084/jem.20061196 PMC2118726

[80] Rosenberg HF , Dyer KD , Foster PS . Eosinophils: changing perspectives in health and disease. Nat Rev Immunol. 2013;13:9–22. 23154224 10.1038/nri3341 PMC4357492

[81] Travers J , Rothenberg ME . Eosinophils in mucosal immune responses. Mucosal Immunol. 2015;8:464–475. 25807184 10.1038/mi.2015.2 PMC4476057

[82] Mesnil C , Raulier S , Paulissen G , et al. Lung‐resident eosinophils represent a distinct regulatory eosinophil subset. J Clin Invest. 2016;126:3279–3295. 27548519 10.1172/JCI85664 PMC5004964

[83] Kupczyk M , Kuna P . Benralizumab: an anti‐IL‐5 receptor α monoclonal antibody in the treatment of asthma. Immunotherapy. 2018:2017–0161. 10.2217/imt-2017-0161 29359607

[84] Wilkerson EM , Johansson MW , Hebert AS , et al. The peripheral blood eosinophil proteome. J Proteome Res. 2016;15:1524–1533. 27005946 10.1021/acs.jproteome.6b00006 PMC5222579

[85] Sturm EM , Schratl P , Schuligoi R , et al. Prostaglandin E2 inhibits eosinophil trafficking through E‐prostanoid 2 receptors. J Immunol. 2008;181:7273–7283. 18981149 10.4049/jimmunol.181.10.7273

[86] Luschnig‐Schratl P , Sturm EM , Konya V , et al. EP4 receptor stimulation down‐regulates human eosinophil function. Cell Mol Life Sci. 2011;68:3573–3587. 21365278 10.1007/s00018-011-0642-5 PMC3192285

[87] Konya V , Sturm EM , Schratl P , et al. Endothelium‐derived prostaglandin I(2) controls the migration of eosinophils. J Allergy Clin Immunol. 2010;125:1105–1113. 20153037 10.1016/j.jaci.2009.12.002

