# Supplementary material for: DP1 receptor signaling prevents the onset of intrinsic apoptosis in eosinophils and functions as a transcriptional modulator
Source: J Leukoc Biol. 2018 Apr 1;104(1):159–71. doi: 10.1002/JLB.3MA1017-404R (PMC6032830; doi:10.1002/JLB.3MA1017-404R)
Supplement: Supplementary file 1 — supplementary information [file JLB-104-159-s001.pdf]

Supplementary Figure 1:

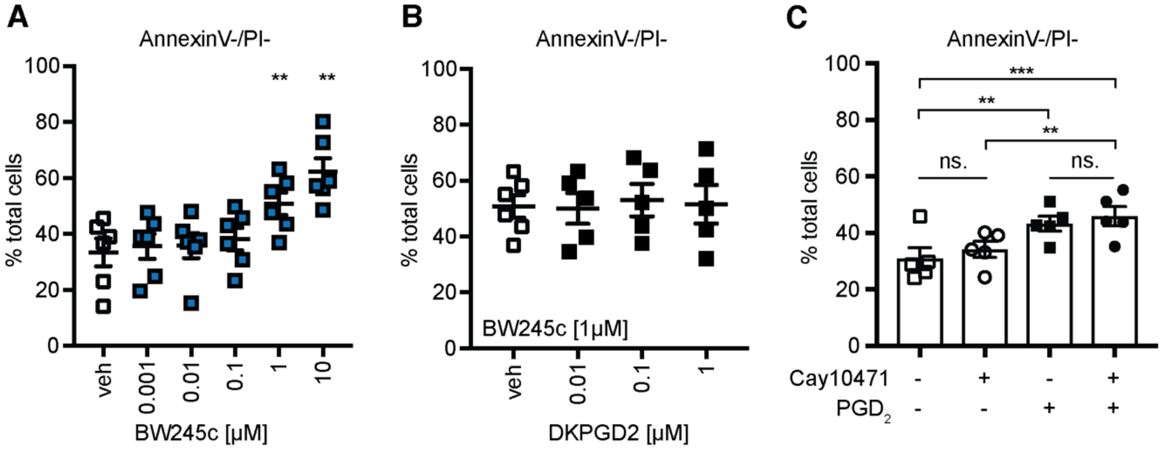

Supplementary Figure 1: **DP1 receptor agonist BW245c promotes survival of eosinophils in a concentration-dependent manner (A). Simultaneous activation of DP2 by DK-PGD_2 does not alter the anti-apoptotic effect of BW245c (B). DP2 antagonist Cay10471 has no significant impact on the capacity of PGD_2 to prolong survival of eosinophils (C).** Isolated eosinophils were cultured with BW245c [0 – 10µM] (A), with 1µM of BW245c and DKPGD2 [0 - 1µM] (B) or were pretreated with vehicle (EtOH) or 1µM of Cay10471 followed by treatment with vehicle (EtOH) or 1µM of PGD_2. A, B and C shows the percentage of annexin V-/PI- cells of total eosinophils. Data show means± SEM of 5-6 individual experiments using eosinophils from different donors.

Supplementary Figure 2:

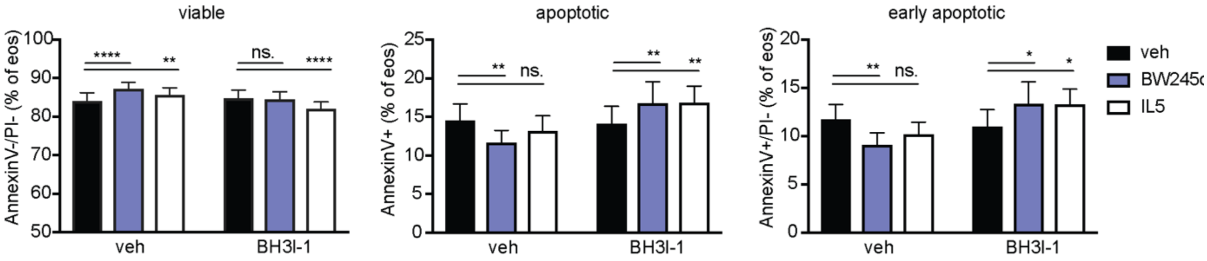

Supplementary Figure 2: **Bcl-2 family protein inhibitor BH3I-1 reverses the anti-apoptotic effect of DP1 receptor activation.** Eosinophils isolated from peripheral blood were incubated with BW245c [1µM] or IL-5 [100pM] in combination with or without Bcl-X_L inhibitor BH3I-1 [50µM] for 5h. BH3I-1 prevented the increase of annexin V-/PI- cells and the reduction of annexin V^+ apoptotic cells induced by BW245c or IL-5. Data show means± SEM of 5 individual experiments.

Supplementary Figure 3:

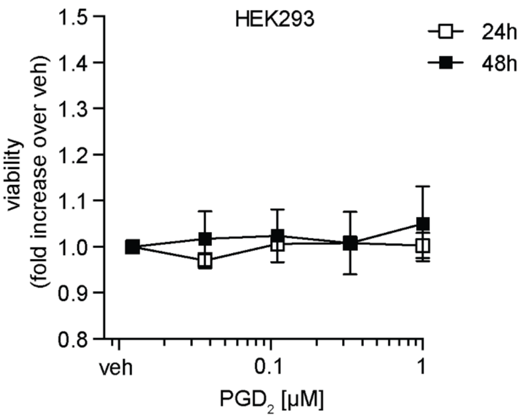

Supplementary Figure 3: **PGD_2 does not alter the viability of parental HEK293 cells in MTS assay.** HEK293 cell lines were starved in OptiMEM for 4h and incubated with increasing concentrations of PGD_2 for 24 or 48h. (n=5)

Supplementary Figure 4:

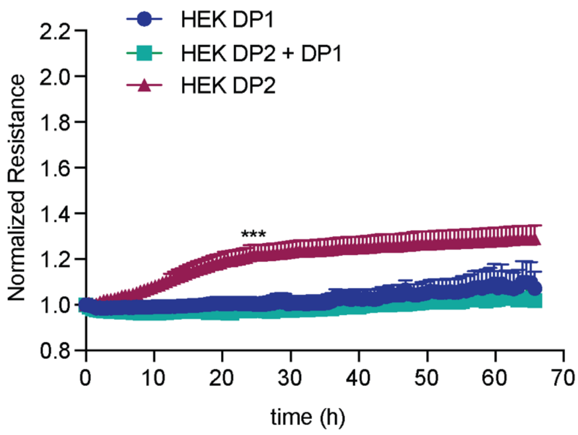

Supplementary Figure 4: **Basal growth of HEK-DP1, HEK-DP2+DP1 and HEK-DP2.** After 15h cultivation in serum free media HEK-DP2 monolayers have a significantly higher resistance than HEK DP1 or HEK DP2+DP2. Electrical resistance was monitored for more than 60 h on a ECIS device. Data show means of 5 independent experiments + SEM.

Supplementary Figure 5:

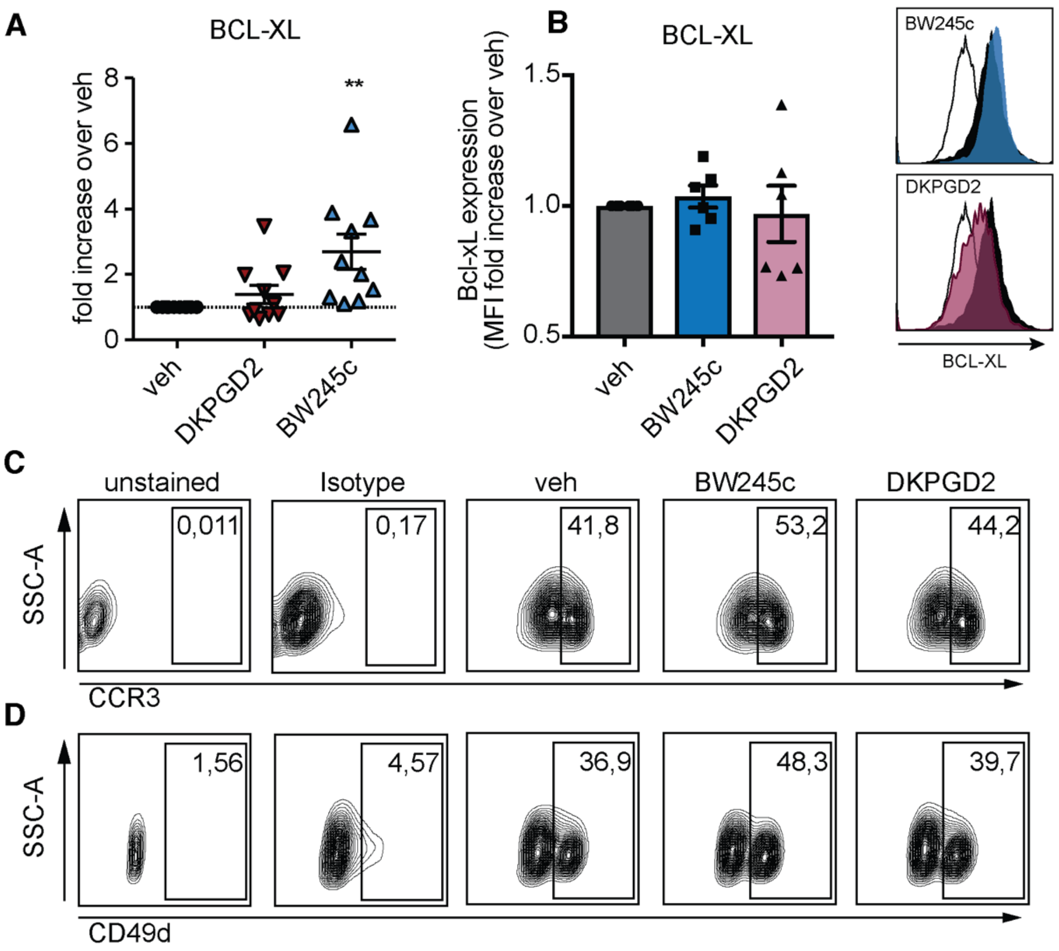

Supplementary Figure 5: **DP1 receptor agonist BW245c enhances (A) mRNA expression of Bcl-X_L (B) but not Bcl-X_L protein level, but enhances CCR3 and CD49d cell surface expression of eosinophils.** Isolated eosinophils ($5x10^6$/ml) were incubated with vehicle (EtOH), DK-PGD_2 [1µM] or BW245c [1µM] for 3 h (A) or 18h (B – D)) in RPMI media containing 1 % FBS and 1%, PenStrep at 37 °C. mRNA expression was measures by qRT PCR (A). (B) Intracellular staining of Bcl-X_L analyzed on a BD FACS Canto flow cytometer. (Histograms: line - isotype control; black – veh; blue – BW245c; red – DKPGD₂) Data show means ± SEM (n=6-10). $^{**}P \leq 0.01$. (C; D) Representative density plots of flow cytometric analyses of CCR3 and CD49d (VLA-4) eosinophil surface expression. Data were acquired on a BD FACS Canto II device.

Supplementary Figure 6:

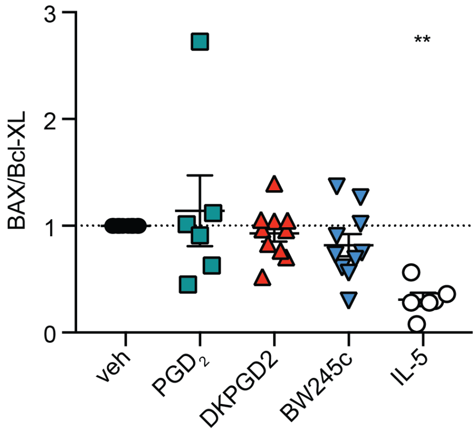

Supplementary Figure 6: **IL-5 decreases ratio of BAX to Bcl-XL mRNA expression as shown by expression of BAX divided by expression of Bcl-X_L.** mRNA expression was measured by qRT PCR. Data show means ± SEM (n=6-10).

Supplementary Figure 7:

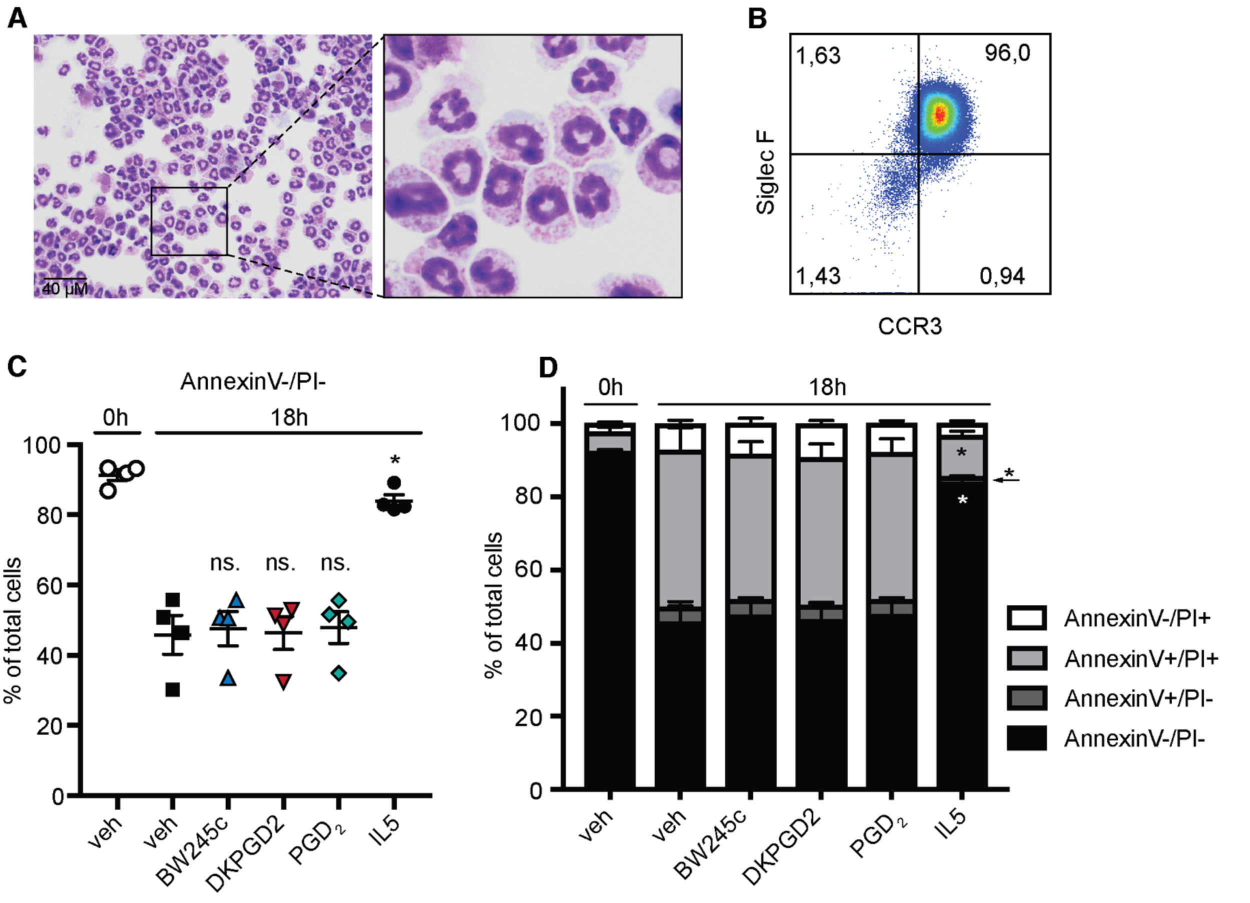

Supplementary Figure 7: **PGD_2 receptor activation does not enhance the survival of murine bone marrow-derived eosinophils (bmEos).** Characterization of bmEos by (A) hematoxylin/eosin staining (A) and flow cytometric analysis of CCR3 and Siglec F expression (B) at day 14. bmEos were cultured with or without 1µM of PGD_2, DK-PGD_2 or BW245c or IL-5 [100 pM] for 18h. BW245c, PGD_2 and DKPGD$_2$ did not significantly enhanced the portion of annexin V^-/PI^- eosinophils as compared to vehicle-treated cells (C). (D) shows the percentage of annexin V^-/PI^-, annexin V^+/PI^-, annexin V^+/PI^+, annexin V^-/PI^+ populations of total bmEos at 18h. Data show means± SEM of 4 bone marrow cultures.

Supplementary Table 1: EC50 values of ligand-induced SRE induction in HEK-DP1, HEK-DP2+DP1 and HEK-DP2 cells.

			HEK-DP1	HEK-DP2+DP1	HEK-DP2
**Agonist** (EC_{50})		**PGD_2**	$2{,}362 \times 10^{-7}$ M	$2{,}474 \times 10^{-7}$ M	-
		**BW245c**	$8{,}368 \times 10^{-9}$ M	$3{,}538 \times 10^{-8}$ M	-
**Antagonist** (IC_{50})	**BWA868c**	$+PGD_2$		$9{,}005 \times 10^{-9}$ M	
	**Cay10471**	$+PGD_2$		$3{,}304 \times 10^{-8}$ M	
	**BWA868c**	+BW245c		$1{,}558 \times 10^{-8}$ M	
	**Cay10471**	+BW245c		$2{,}571 \times 10^{-9}$ M	

Data are means ± SEMs of 3 to 6 independent experiments. EC_{50}, half maximal effective concentration; IC_{50}, half maximal inhibitory concentration; -, no response

Supplementary Methods:

Flow cytometric analysis of Bcl-XL expression

Isolated eosinophils (5×10^5/ml) were kept in RPMI (Thermo Fisher Scientific) supplemented with 1% FBS and PenStrep (Sigma-Aldrich) and stimulated with 1 µM DK PGD_2 or BW245c for 18h at 37°C. Cells were fixed (2 % formaldehyde) and permeabilized (0.1 % Triton X) prior to staining with primary rabbit anti-human-BcL-xL (Cell signaling, 54H6) or isotype control, and secondary goat anti-rabbit Alexa Flour 488 (Life Technologies). Data were acquired on a BD FACS Calibur.

Generation of bone-marrow derived eosinophils (bmEos)

Bone marrow cells of wild-type Balb/c mice were differentiated *ex vivo* into bmEos as previously described.[1] At day 14 bmEos were characterized by flow cytometric analysis of CCR3 (rat anti-mouse CCR3-FITC, R&D systems, MN, USA) and Siglec F (rat anti-mouse Siglec F-PE, BD Biosciences, NJ, USA) expression and by staining cytospins with Hemacolor stain (Millipore, MA; USA). The purity of bmEos at day 14 was > 90%.

Reference:

1 Dyer KD, Moser JM, Czapiga M, Siegel SJ, Percopo CM, Helene F *et al.* Functionally competent eosinophis differentiated ex vivo in high purity from normal mouse bone marrow. *J Immunol* 2008; **181**: 4004–4009.
